# Bivalent RSV prefusion F vaccination elicits effective neutralization of contemporary and monoclonal antibody-resistant RSV strains

**DOI:** 10.1038/s41541-026-01418-8

**Published:** 2026-03-14

**Authors:** Wei Chen, Lyndsey T. Martinez, Larissa Falcao, Zhenghui Li, Andrew P. McKeen, Chaitanya Kurhade, Hélène Boigard, Vidia Roopchand, Imani Richardson, Trisha Dasgupta, Katrina E. Llamera, Jing Colatat, Linda Goding Brock, Annaliesa S. Anderson, Kena A. Swanson

**Affiliations:** 1 Vaccines, Pfizer Inc, Pearl River, NY USA; 2 Data Sciences and Analytics, Pfizer Inc, Pearl River, NY USA; 3 Present Address: Regeneron Pharmaceuticals, Tarrytown, NY USA

**Keywords:** Diseases, Immunology, Microbiology

## Abstract

Respiratory syncytial virus (RSV) is the leading global cause of serious respiratory disease in infants and an important respiratory pathogen in older adults. The RSV prefusion F protein (preF) is a major target of neutralizing antibodies shown to protect against RSV disease. The bivalent preF protein subunit vaccine (RSVpreF; Abrysvo®) contains stabilized preF antigens representing the two major RSV subgroups, RSV A and RSV B. Here, we characterized the neutralizing activity of adult RSVpreF immune sera against a panel of 65 contemporary, globally circulating RSV A and RSV B clinical isolates, containing various amino acid substitutions across the five major antigenic sites of RSV F (Ø, I, II, III, V). Monoclonal Ab-resistant mutant strains (MARMs) displaying in vitro resistance to nirsevimab, clesrovimab, and palivizumab (up to 300,000-fold resistance over the parental strain) were also evaluated. RSVpreF immune sera effectively neutralized both the panel of global clinical isolates and all MARMs tested. These findings demonstrate that the bivalent RSVpreF polyclonal response maintains robust neutralizing activity against circulating RSV A and B strains, including those that escape RSV F mAbs, and provides broad protective immunity against RSV.

## Introduction

Human respiratory syncytial virus (RSV) is a common respiratory virus and major cause of severe lower respiratory tract illness in infants^1^, older adults, especially those aged ≥65 years, as well as adults with chronic cardiovascular or lung disease, or immunocompromising conditions^2,3^. RSV vaccines and two next-generation monoclonal antibody (mAb) were recently licensed for prevention of RSV disease. The bivalent RSVpreF (Abrysvo®) vaccine was approved in 2023 and is indicated for maternal immunization for protection of infants, in addition to active immunization of adults 60 years and older and high-risk individuals 18-59 years of age for protection against RSV lower respiratory tract disease (LRTD). In clinical trials, RSVpreF elicited robust neutralizing responses against RSV A and RSV B in adults^4–8^ and pregnant women, the latter whose antibodies were efficiently transplacentally transferred to their infants^9^ and was efficacious^10–13^. Pivotal efficacy studies with preF-specific mAbs have also demonstrated significant reduction of RSV disease in infants. Nirsevimab (MEDI-8897; Beyfortus®)^14^ was approved in 2023 in the US for use in newborns and infants in their first RSV season and at-risk children up to 24 months of age in their second RSV season^15^, and clesrovimab (MK-1654; Rb1; Enflonsia™) was approved in 2025 for the prevention of RSV LRTD in infants born during or entering their first RSV season^16^.

RSV is an enveloped negative-sense, single-stranded RNA virus and has two major antigenic subgroups, A and B, that co-circulate; both are associated with severe RSV disease^17,18^. The primary antigenic determinant for the RSV subgroups is the G glycoprotein^19,20^; however, the F glycoprotein that mediates virus entry is the major antigenic target for prophylactic vaccines and mAbs^21^. RSV F exists as a trimer of heterodimers (covalently-linked F1 and F2 subunits) in an active, metastable prefusion conformation that transitions to a highly stable postfusion state while mediating fusion of viral and host cell membranes for virus entry^22,23^. In contrast to RSV G, the mature F protein has only three N-linked glycosylation sites per protomer (N27, N70, and N500)^24,25^ and is more conserved, with 90% amino acid (aa) sequence identity between RSV A and B subgroups.

The most potent neutralizing antibodies specific to RSV F trimer recognize the prefusion conformation, where five major antigenic sites have been identified: Ø, II, III, IV, and V^21,26–28^. Sites Ø and V, found on the apex, or crown, of the trimer are the most sensitive to antibody neutralization and are restricted to the prefusion F (preF) conformation^29,30^. Site III mAbs preferably bind preF^31^; sites I, II, and IV are shared between the prefusion and postfusion F conformations, with site I being the least sensitive to neutralization^32^. RSV F protein sequence variability in surface-exposed residues between RSV A and B subgroup viruses cluster in prefusion-specific antigenic sites, such as site Ø^23,33,34^. Virus surveillance studies have shown that subgroup B viruses tend to have more dynamic non-synonymous sequence changes, particularly in site Ø, whereas RSV A site Ø sequences have remained largely stable^34–37^.

The potential of RSV F neutralizing antibodies to protect against RSV disease was first established with palivizumab (Synagis®), a prophylactic humanized RSV F mAb, that demonstrated prevention of RSV-associated hospitalizations in high-risk infants^38^. Epitope specificity is an important determinant of RSV mAb neutralizing potency, as nirsevimab, which targets site Ø^21^, was shown to provide superior protection against RSV in vivo over the murine precursor of palivizumab^39^, which binds site II. Clesrovimab, discovered after nirsevimab, binds site IV^40^. Both nirsevimab and clesrovimab contain mutations in three aa positions (M252Y/S254T/T256E [YTE]) in the Fc region to support an extended antibody half-life^41,42^. Other mAb prophylaxes for RSV disease, including suptavumab and motavizumab, targeting site IV and II, respectively^43,44^, were developed but demonstrated unfavorable results in clinical trials^44,45^. Suptavumab’s phase 3 trial did not meet its primary efficacy endpoint due to neutralization escape of RSV B strains circulating during the trial harboring a naturally occurring mutation in the suptavumab binding site^44^. Similarly, under selective pressure in vitro, RSV can escape mAb-based neutralization, and nirsevimab and clesrovimab mAb-resistant strains have been previously generated for both subgroups^40,46^.

One of the major considerations for an effective RSV vaccine is its ability to elicit antibodies capable of neutralizing a broad array of RSV strains. The breadth of vaccine-elicited neutralization responses against select RSV strains was previously characterized using the monovalent RSVpreF3-AS01 vaccine (Arexvy®)^47^. Here, we evaluated the neutralization breadth of the bivalent RSVpreF vaccine against an extensive panel of contemporary and antigenically diverse RSV A and B strains. We sought to understand F protein sequence diversity over several RSV seasons prior to the introduction of RSV vaccines and mAbs in 2023. Adult immune sera from the phase 1/2 first-in-human randomized study of RSVpreF^4^, in addition to the prophylactic RSV mAbs nirsevimab, clesrovimab, and palivizumab, were assessed for their ability to neutralize RSV clinical isolates and over two dozen monoclonal antibody resistant mutant strains (MARMs).

## Results

### RSV F genotypic analysis of contemporary RSV A and RSV B clinical isolates

A contemporary and geographically diverse panel of RSV A and RSV B clinical isolates, most with a unique F protein aa sequence (i.e., aa substitution or set of aa substitutions not found in other isolates) that differed from the RSVpreF F sequences, were first characterized by whole genome sequencing (WGS) (Supplementary Fig. 1). Ontario and Buenos Aires were confirmed to be the predominant RSV A and RSV B genotypes, respectively^48,49^. WGS analysis was further used to identify isolates bearing aa polymorphisms in the full-length F sequence, relative to the bivalent RSVpreF vaccine antigen sequences (also based on the Ontario and Buenos Aires genotypes). A total of 65 RSV isolates were selected: 42 RSV A and 23 RSV B isolates that contained one or more naturally occurring aa substitutions in the F protein. Viruses were isolated in five countries from four continents (South America, North America, Europe, and Africa) (Supplementary Fig. 2A) across seven RSV seasons: 2015 to 2023 in the southern hemisphere (SH), and the 2015/16 to 2023/24 season in the northern hemisphere (NH) (Supplementary Fig. 2B). Greater than 30% of selected RSV A and B isolates were from South Africa and Spain, respectively. Most isolates were collected during the 2023 SH and 2022/23 NH RSV seasons, which represented roughly one-third and one-half of total RSV isolates from SH and NH seasons, respectively.

Compared to the RSV A preF vaccine antigen sequence, antigenic site changes were detected in six out of 42 RSV A isolates: PFERSV335 and PFERSV400 contained A74T or N63S in site Ø; PFERSV333, PFERSV421, PFERSV425 contained S276N in site II; and PFERSV408 contained S173Y in site V (Table 1, Supplementary Fig. 3). A74T and N63S substitutions were observed at very low prevalence (0.39 and 0.16%, respectively) in RSV A sequences deposited in GISAID through the end of 2024; S276N was detected in 5.2% of RSV A sequences; and site V substitutions (S173Y/L/T) were present in less than 0.1% of RSV A sequences (Supplementary Table 1). All 23 RSV B isolate sequences deposited in GISAID through the end of 2024 contained aa substitutions in one or multiple antigenic sites (Ø, I, II, III or V), including F45L in site III, and L172Q and S173L in site V (Table 2), which are not found in the RSV B F sequence of RSVpreF. F45L/L172Q/S173L were found in 31% of the RSV B sequences deposited in GISAID as of 2024. This is consistent with a prior report that found L172Q/S173L was predominant among circulating RSV B strains in the US by 2017^36^. In addition, all RSV B isolates since the 2017/18 RSV season contained additional substitutions, I206M and Q209R, in site Ø. Isolates collected after 2021 (Spain) or 2022 (South Africa) RSV seasons contained an additional new site Ø substitution, S211N. The site I S389P substitution, first detected in the 2021/22 RSV season, was consistently found in combination with site Ø substitutions I206M, Q209R, and S211N in isolates of this collection (Table 2, Supplementary Fig. 3). Site II S276N/I was found in two isolates (PFERSV297 and PFERSV391). Unlike the low prevalence of aa substitutions in RSV A strains, RSV B entries with a combination of antigenic site mutations (F45L/L172Q/S173L/I206M/Q209R) represented 22.15% of total RSV B sequences deposited in GISAID during the same period (Supplementary Table 1).

**Table 1:** RSV F protein amino acid substitutions in RSV A viral clinical isolates in circulation between 2015 and 2023

Country of Isolation	Clinical isolate ID	RSV Season	Passage No.	Amino acid change(s) on F protein (Antigenic site, if affected)
South Africa	PFERSV004	2016	2	A23T
	PFERSV005		1	A23T, T528I
	PFERSV007			None
	PFERSV010	2015	3	A23T, T234A
	PFERSV400	2023	2	N63S (**Site Ø**), T122A
	PFERSV401			S105N, T122A
	PFERSV402			T12I
	PFERSV403			A23S, A107T, T122A
	PFERSV404			T122A
	PFERSV406			N9K, T122A
	PFERSV407			A23S, K42R, T122A
	PFERSV408	2019	2	S173Y (**Site V**)
	PFERSV411			L3S, T122A, K123Q, I384T
	PFERSV413			T122A, K123Q, I384T, S554N
	PFERSV414			T122A, K123Q, I384T
United States	PFERSV011	2016–2017	Original	None
	PFERSV017			T100A
	PFERSV025			R553K
	PFERSV027			F22S, T125A
	PFERSV333	2018–2019	2	T12I, A23T, S276N (**Site II**)
	PFERSV334	2019–2020		None
	PFERSV335			A23T, A74T (**Site Ø**)
	PFERSV352	2022–2023		T12I, F114Y
	PFERSV353			T13A, N124T
	PFERSV354			I57V, V76A, L111I, T122A
	PFERSV358			A103T, T122A, V533I
Chile	PFERSV266	2015	Original	None
	PFERSV274	2017		L119I, S466N
The Netherlands	PFERSV302	2015–2016	Original	None
	PFERSV303			N120S
	PFERSV305	2017–2018		A102V, S105N
	PFERSV306			E2D, A518V
	PFERSV307			F114S, T555A
Spain	PFERSV417	2022–2023	2	P4S
	PFERSV418			T12I, M115I
	PFERSV419			A23V, V76A, A103T, T122A
	PFERSV420			A23V, A103T, T122A
	PFERSV421			T12I, L20F, S276N (**Site II**), I524T
	PFERSV423			A103T, T122A
	PFERSV425			T12I, L20F, V76I, S276N (**Site II**)
	PFERSV426			A103T, T122A, V379A
	PFERSV427			A103T, T122A, V406I

Amino acid changes identified in the F protein sequences of RSV clinical isolates (*n* = 42) respective to the RSVA/*Homo sapiens*/USA/LA2_21/2013 reference strain.

**Table 2:** RSV F protein amino acid substitutions in RSV B clinical isolates collected between 2015 and 2023

Country of Isolation	Clinical isolate ID	RSV Season	Passage No.	Amino acid change(s) on F protein (Antigenic Ø, if affected)
United States	PFERSV012	2016–2017	Original	F45L (Site III), A103V, L172Q (Site V), S173L (Site V)
	PFERSV014			L22P, F45L(Site III), A103V, L172Q(Site V), S173L (Site V)
	PFERSV018			A19T, F45L(Site III), A103V, L172Q(Site V), S173L (Site V)
	PFERSV020			F45L(Site III), A103V, L172Q(Site V), S173L(Site V), A529V
	PFERSV022			F45L(Site III), A103V, L172Q(Site V), S173L(Site V), S409A
Chile	PFERSV277	2018	Original	F45L(Site III), A103V, L172Q(Site V), S173L(Site V), K191R, I206M (Site Ø), Q209R (Site Ø)
The Netherlands	PFERSV292	2015–2016	Original	L22P, F45L(Site III), A103V, M115I, L172Q(Site V), S173L(Site V)
	PFERSV293			F45L(Site III), A103V, L172Q(Site V), S173L (Site V)
	PFERSV294	2016–2017		L22P, F45L(Site III), A103V, L172Q(Site V), S173L(Site V), L462Q
	PFERSV297	2017–2018		F12L, F45L(Site III), A103V, L172Q(Site V), S173L(Site V), K191R, I206M (Site Ø), Q209R (Site Ø), S276N (Site II), E463D
	PFERSV301			F45L(Site III), A103V, L172Q(Site V), S173L(Site V), K191R, I206M (Site Ø), Q209R (Site Ø)
South Africa	PFERSV380	2022	2	F45L(Site III), A103V, N120S, L172Q(Site V), S173L(Site V), K191R, I206M (Site Ø), Q209R (Site Ø)
	PFERSV381			F45L(Site III), A103V, L172Q(Site V), S173L(Site V), K191R, I206M (Site Ø), Q209R (Site Ø)
	PFERSV386	2023		A19T, F45L(Site III), A103V, L172Q(Site V), S173L(Site V), K191R, I206M (Site Ø), Q209R (Site Ø), S211N(Site Ø), S389P (Site I)
	PFERSV387	2019		F45L(Site III), A103V, L172Q(Site V), S173L(Site V), K191R,I206M (Site Ø), Q209R (Site Ø), I525V
	PFERSV389	2019		F45L(Site III), A103V, L172Q(Site V), S173L(Site V), K191R, I206M (Site Ø), Q209R (Site Ø)
Spain	PFERSV390	2021–2022		L4P, F12L, F45L(Site III), A103V, L172Q(Site V), S173L(Site V), S190N, K191R, I206M (Site Ø), Q209R (Site Ø), S211N(Site Ø), S389P (Site I)
	PFERSV391	2022–2023		F45L(Site III), A103V, L172Q(Site V), S173L(Site V), S190N, K191R, I206M (Site Ø), Q209R (Site Ø), S211N(Site Ø), S276I (Site II), S389P (Site I)
	PFERSV393			R42K, F45L(Site III), A103V, L172Q(Site V), S173L(Site V), S190N, K191R, I206M (Site Ø), Q209R (Site Ø), S211N(Site Ø), S389P (Site I)
	PFERSV395			R42K, F45L(Site III), A103V, L172Q(Site V), S173L(Site V), S190N, K191R, I206M (Site Ø), Q209R (Site Ø), S211N(Site Ø)
	PFERSV396			F45L(Site III), A103V, N116S, L172Q(Site V), S173L(Site V), S190N, K191R, I206M (Site Ø), Q209R (Site Ø), S211N(Site Ø), S389P (Site I)
	PFERSV397			F45L(Site III), A103V, L172Q(Site V), S173L(Site V), S190N, K191R, I206M (Site Ø), Q209R (Site Ø), S211N(Site Ø), S389P (Site I)
	PFERSV399			F12I, F45L(Site III), A103V, L172Q(Site V), S173L(Site V), S190N, K191R, I206M (Site Ø), Q209R (Site Ø), S211N(Site Ø), S389P (Site I)

Amino acid changes in the F protein sequences of RSV clinical isolates (*n* = 23) respective to the RSVB/*Homo sapiens*/PER/FPP00592/2011 reference strain.

### Bivalent RSVpreF immunization boosted neutralizing antibody titers against contemporary RSV A and B clinical isolates

**Fig. 1:**
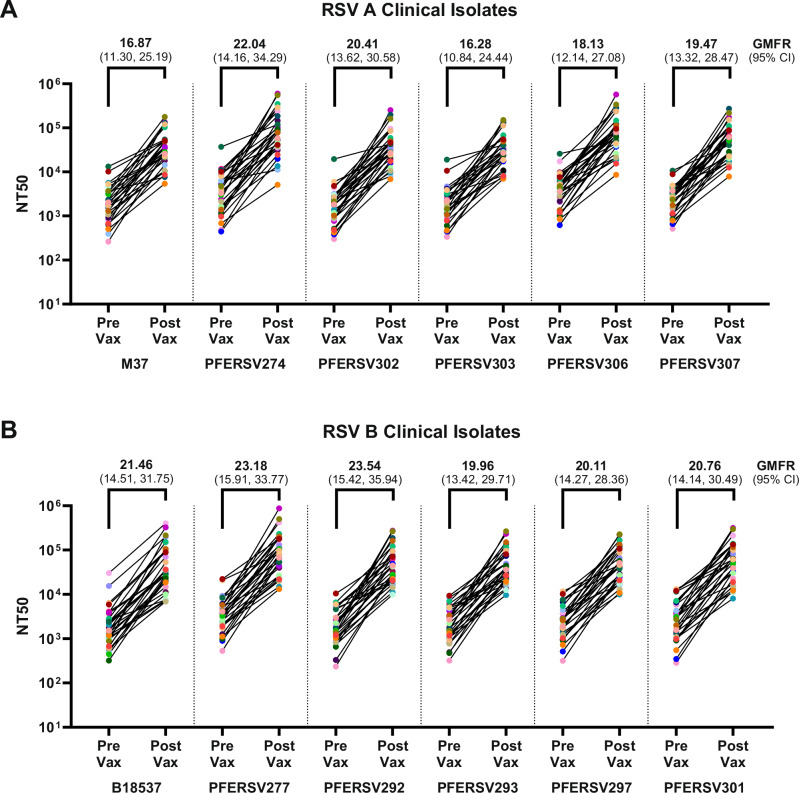
RSVpreF vaccination boosted RSV A and RSV B neutralization antibody levels. Human sera from trial participants pre- and post- vaccination with 120 μg dose of RSVpreF from the Phase 1/2 clinical study (NCT03529773) (*n* = 30) were tested for their neutralizing activities against selected (**A**) RSV A clinical isolates (PFERSV274, PFERSV302, PFERSV303 PFERSV306, PFERSV307) and (**B**) RSV B clinical isolates (PFERSV277, PFERSV292, PFERSV293, PFERSV297, PFERSV301) in the RSV non-clinical neutralization assays. M37 and B18537 served as reference strains for RSV A and B, respectively. 50% virus neutralizing titers (NT_50_) are represented by a colored dot for individual sera, with lines connecting the pre- and post-vaccination titers for each participant. Additionally, the geometric mean fold rise (GMFR) in neutralizing titer with 95% confidence interval (in parentheses) is shown above the individual sera data, for each isolate tested.

**Fig. 2:**
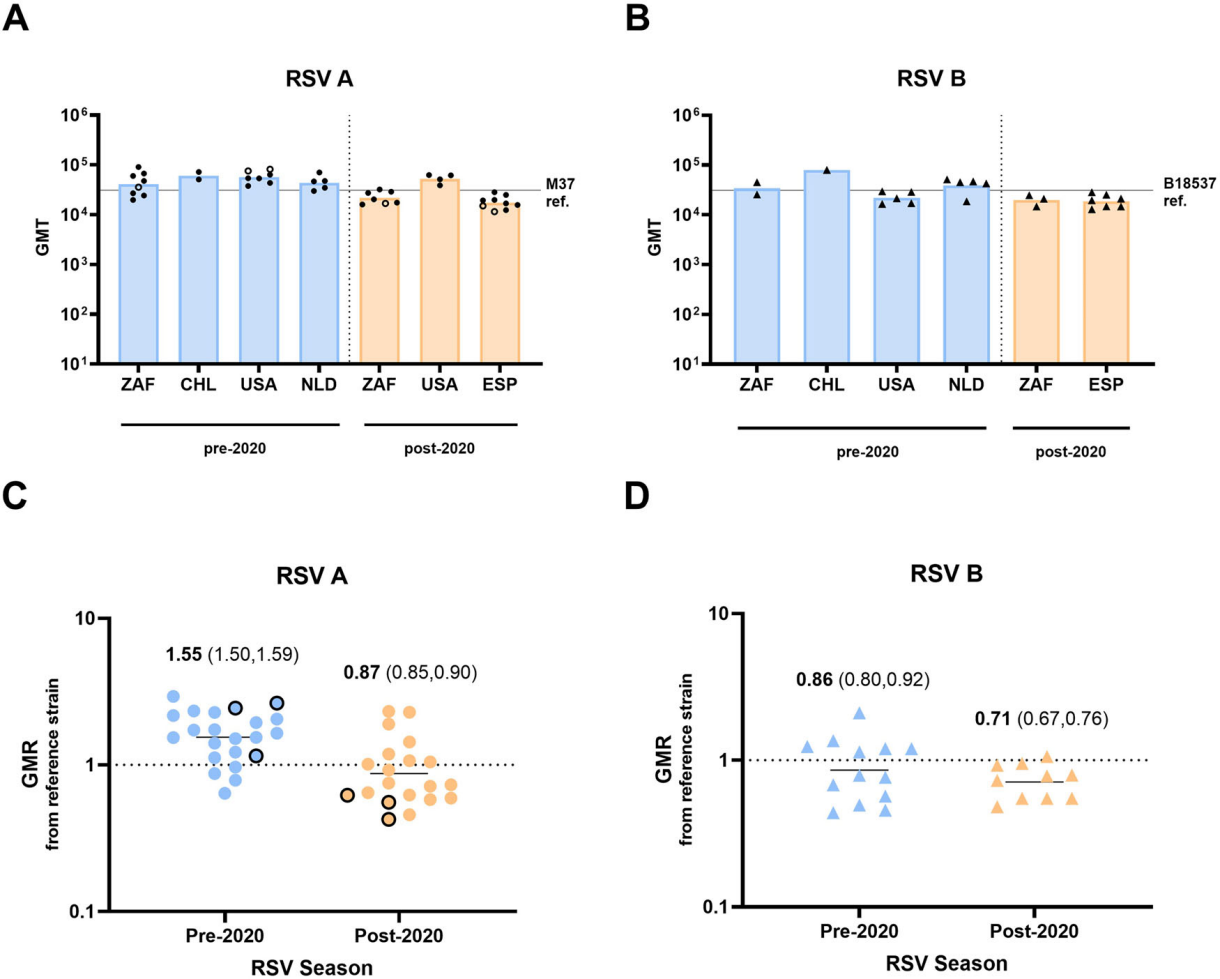
RSVpreF immune sera effectively neutralized global circulating clinical RSV A and RSV B strains from pre- and post-2020 RSV seasons. Adult immune sera collected one month after vaccination with a single dose of RSVpreF (NCT03529773) (*n* = 30) were tested against a panel of (**A**) RSV A clinical isolates from pre-(*n* = 22) and post-2020 (*n* = 20) RSV seasons, and (**B**) RSV B clinical isolates collected pre- (*n* = 13) and post-2020 (*n* = 10) RSV seasons. Pre-2020 and post-2020 isolates are separated by a vertical dotted line. Each symbol in (**A**) and (**B**) represents the geometric mean 50% virus neutralizing titer, or geometric mean titer (GMT), per clinical isolate. Bars represent the geometric mean of isolate GMTs per country. GMT values for the M37 and B18537 reference strains are shown as solid lines. RSVpreF immune sera neutralization expressed as the geometric mean ratio (GMR, bold values) for each (**C**) RSV A and (**D**) RSV B clinical isolate, from the reference strain, with 95% CIs (values shown in parentheses). The open circles in (**A**) and black outlined circles in (**C**) represent the six RSV A isolates with aa substitutions in at least one RSV F antigenic site. All RSV B isolates contained aa substitutions in at least one RSV F antigenic site. In **C** and **D**, the dotted line at *y* = 1 indicates no fold change and black lines indicate GMT for each group of pre- or -post-2020 isolates. ZAF, South Africa; CHL. Chile; NLD, the Netherlands; ESP, Spain.

Based on the WGS analysis, the 42 RSV A isolates and 23 RSV B isolates identified with RSV F protein aa substitutions were assayed for neutralization susceptibility to RSVpreF immune sera. In prior clinical trials in healthy adults aged 18–49, RSVpreF vaccination has been reported to boost pre-existing RSV A and RSV B immunity 12- and 14-fold, respectively, as compared to pre-vaccinated levels against reference strains M37 (RSV A) and B18537 (RSV B)^4^. Using a subset of human pre- and 1-month post-vaccination RSVpreF immune sera from the prior phase 1/2 study (NCT03529773), conducted from 2018 through 2020, we found similarly robust increases in RSV A and RSV B neutralization titers against both the reference strains and randomly selected clinical RSV A and RSV B (*n* = 5 each) isolates tested; 16- to 24-times higher neutralizing geometric mean titers (GMTs) were observed relative to individual baseline titers against M37 and B18537 [geometric mean fold rise (GMFR) 16.87 and 21.46, respectively] and the ten isolates (GMFR range: 16.28–23.54) (Fig. 1A and B). Neutralizing GMTs for RSV clinical isolates collected from various countries prior to (RSV A: *n* = 22; RSV B: *n* = 13) and after (RSV A: *n* = 20; RSV B: *n* = 10) the start of the COVID-19 pandemic (2020) trended slightly higher or similar to that of M37 (Fig. 2A) and B18537 (Fig. 2B). Over eight RSV seasons, the combined GMT of all individual isolate GMTs was 33,968 and 26,090 for RSV A and RSV B (Supplementary Tables 2 and 3), respectively, indicating comparable vaccine-elicited neutralizing titers against unique RSV A and B subgroup isolates (Supplementary Fig. 4). RSVpreF neutralizing activity was generally similar among RSV A isolates (open circles in Fig. 2A, black outlined circles in Fig. 2C), and RSV B isolates with aa substitutions localized to site Ø, II or V compared to the corresponding reference strain. Average geometric mean ratio (GMRs) for pre-2020 and post-2020 isolates relative to the reference strain were close to 1 (1.55 [1.50,1.59 95% CI] and 0.87 [0.85,0.90 95% CI] for RSV A, Fig. 2C; 0.86 [0.80,0.92 95% CI] and 0.71 [0.67,0.76 95% CI] for RSV B, Fig. 2D). The lower bounds of the 95% CI adjusted GMRs for pre-and post-2020 isolates were above 0.667^8^, suggesting neutralizing activity comparable to the respective reference strain. The complete one-month post-vaccination GMRs for all RSV A and RSV B isolates (relative to their respective reference strains) are listed in Supplementary Tables 2 and 3, respectively. Individual vaccinee neutralizing GMTs per clinical isolate are graphed in Supplementary Fig. 4. Overall, these findings demonstrate that adult RSVpreF immune sera effectively neutralized contemporary RSV A and B isolates in comparison to reference strains, regardless of aa substitutions in F antigenic sites or country of origin.

### Differential nirsevimab neutralization of RSV A and RSV B strains

We next sought to compare the ability of RSV mAbs, which target a single epitope on RSV F, and RSVpreF polyclonal immune sera to neutralize selected RSV A and B clinical isolates. Half-maximal inhibitory concentrations (IC_50_) were calculated using an RSV neutralization assay to first characterize potential nirsevimab resistance against select RSV clinical isolates with antigenic site Ø substitutions [RSV A (*n* = 5) and RSV B (*n* = 9)] (Supplementary Table 4). Having first emerged in the 2017-18 RSV season and now endemic since 2019, at least 94% of RSV B genomes contain the prevalent nirsevimab binding site substitutions I206M + Q209R in the F antigenic site Ø (Supplementary Table 5). Thus, we chose to focus on the effect of this double polymorphism on nirsevimab resistance, and RSV B isolates with and without I206M + Q209R were selected at random for neutralization testing.

**Fig. 3:**
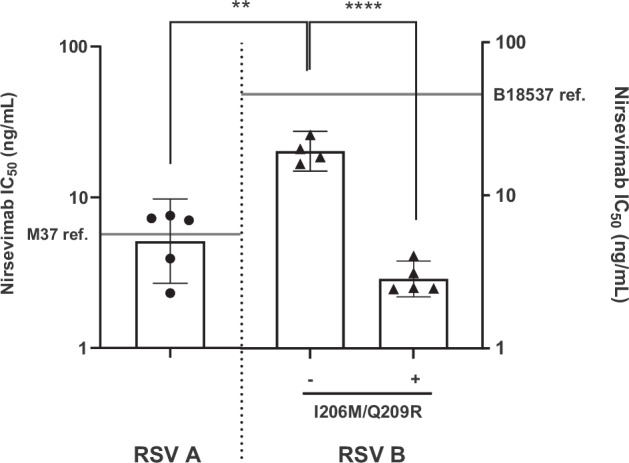
Susceptibility of RSV A and RSV B clinical isolates to nirsevimab. Nirsevimab (commercial Beyfortus®) was tested for neutralization activity against selected RSV clinical isolates with F protein antigenic site substitutions. Nirsevimab IC_50_ (half-maximal inhibitory y concentration) against select RSV A (*n* = 5, circles) and RSV B clinical isolates (*n* = 9, triangles) are shown. In RSV B isolates, the I206M/Q209R double substitution in RSV F site Ø was either absent (*n* = 4, pre-2017/18 RSV season) or present (*n* = 5, 2017/18 RSV season and onward). Bars represent geometric mean concentration (GMC), and error bars represent 95% CI. IC_50_ values for the M37 and B18537 reference strains are shown as solid lines for comparison. Table S4 provides the complete list of IC_50_ values for each isolate and reference strain tested. Statistical significance was determined by an ANOVA and adjusted using a Bonferroni multiple comparisons test. **p < 0.01, ****p < 0.0001.

Nirsevimab’s IC_50_ was 5.7 ng/mL and 45.8 ng/mL for the reference strains M37 and B18537, respectively, demonstrating an 8.0-fold lower sensitivity to nirsevimab for RSV B than RSV A (Fig. 3, Supplementary Table 4). This contrasts with a prior study, where RSV A and B reference strains, MI-A2 and B9320, had similar nirsevimab IC_50_ values^46^. Pairwise amino acid sequence identity of F between B9320 and B18537 is 99%, differing in just two amino acid residues, S197N and Q202R, at the nirsevimab binding site. The IC_50_ GMC (Geometric Mean Concentration) of the RSV A isolates was 5.1 ng/mL, signifying that RSV A susceptibility to nirsevimab was comparable to that of M37. All RSV B isolates tested, regardless of site Ø substitutions, were more susceptible to nirsevimab neutralization as compared to B18537. RSV B isolates lacking the I206M + Q209R polymorphism (*n* = 4) had an IC_50_ GMC of 19.4 ng/mL, demonstrating a significantly lower susceptibility to nirsevimab neutralization as compared to the five RSV A isolates (*p* = 0.005), and a significantly lower susceptibility as compared to the five RSV B isolates with I206M + Q209R (IC_50_ GMC 2.8 ng/mL, *p* < 0.0001) (Fig. 3, Supplementary Table 4). Thus, among the isolates tested, RSV B isolates lacking the I206M + Q209R substitution were the least susceptible to nirsevimab neutralization, while RSV B isolates containing I206M + Q209R were more susceptible, consistent with a prior report^50^.

In this study, neutralization sensitivities of M37 and B18537 towards nirsevimab were also confirmed using a previously described validated clinical RSV neutralization assay^51^. To determine 50% neutralizing antibody titers that correspond to nirsevimab protective concentrations, nirsevimab (Beyfortus) was spiked into pooled, unvaccinated adult human sera to achieve concentrations of 6.8 µg/mL, the 90% effective threshold previously established through preclinical and clinical studies^52^, 3.4 µg/mL (2-fold lower), and up to 13.6 µg/mL (2-fold higher). There was a marginally significant (*p* = 0.08) effect between subgroup and nirsevimab concentration, when evaluated using an ANOVA, with a significant difference observed at 13.6 µg /mL nirsevimab (*p* = 0.007) (Supplementary Fig. 5). Nonetheless, nirsevimab-spiked sera showed lower neutralizing activity against B18537 compared to M37 at all four nirsevimab concentrations, including at the established protective threshold of 6.8 µg/mL, indicating that, for these reference strains, RSV B is less effectively neutralized than RSV A at clinically relevant concentrations of nirsevimab.

### Bivalent RSVpreF immune sera neutralize RSV monoclonal antibody-resistant mutants (MARMs)

**Fig. 4:**
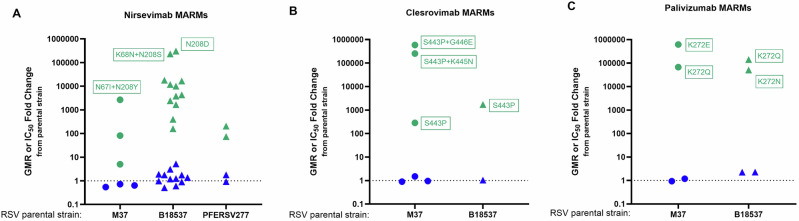
RSVpreF immune sera neutralizes nirsevimab-, clesrovimab-, and palivizumab-resistant RSV A and RSV B strains. Several monoclonal antibody (mAb)-resistant strains (MARMs) were generated recombinantly from reference strains M37 (RSV A) or B18537 (RSV B) and analyzed for their resistance to the corresponding pressure mAb: **A** nirsevimab, **B** clesrovimab, or **C** palivizumab. Two additional nirsevimab-resistant strains were generated in vitro on the RSV B clinical isolate, PFERSV277, under nirsevimab pressure. Nirsevimab, clesrovimab, or palivizumab MARMs were also assayed for their resistance to RSVpreF immune sera from vaccinated participants (*n* = 30) (NCT03529773). mAb (green symbols) and RSVpreF immune sera (blue symbols) neutralization resistance are expressed as fold change in IC_50_ or geometric mean ratio (GMR), respectively, for each nirsevimab-resistant-, clesrovimab-resistant-, and palivizumab-resistant-strain, over the parental strain (see Table 3). Each MARM is represented by a single symbol (RSV A, circles; RSV B, triangles). The dashed line at *y* = 1 indicates no fold change. Amino acid position changes of specific MARMs are indicated in adjacent text boxes.

To further assess the neutralizing ability of RSVpreF immune sera versus RSV F-specific prophylactic mAbs, RSV MARMs were generated recombinantly using reverse genetics or by repeated exposure of RSV B18537 and the RSV B clinical isolate PFERSV277 to the mAb in vitro. Over two dozen MARMs were generated and tested against nirsevimab (*n* = 17) (Fig. 4A), clesrovimab (*n* = 4) (Fig. 4B), or palivizumab (*n* = 4) (Fig. 4C). Four RSV B MARMs in this study contained newly identified substitutions (S62R, K68I, L203V, L204I), while the remainder (84%) were of both subgroups and representative of prior reports^40,46,50,53–55^. MARMs evaluated in this study demonstrated resistance by requiring higher concentrations of each mAb (IC_50_) to effectively neutralize the virus relative to the parental strain (Fig. 4A–C). For nirsevimab, the RSV B MARM with a N208D substitution in F had the highest fold resistance (IC_50_ 306,465 times higher than parental B18537 strain); for clesrovimab, the RSV A MARM with a S443P + G446E substitution was most resistant to neutralization (IC_50_ > 586,667 times higher than parental M37 strain); for palivizumab, the RSV A MARM with a K272E substitution was most resistant to neutralization (IC_50_ > 634,518 times higher than parental M37 strain) (Table 3, Fig. 4C). To evaluate MARMs with multiple antigenic site substitutions, PFERSV277, containing substitutions in sites Ø (I206M + Q209R), III (F45L), and V (L172Q, S173L), was propagated under nirsevimab selective pressure. WGS confirmed additional K65E and K68I substitutions, both in the antigenic site Ø, were separately introduced into two PFERSV277-based MARMs. Each MARM demonstrated 203 and 75-fold increased resistance to nirsevimab, respectively, as compared to the parental PFERSV277 strain (Fig. 4A, Table 3).

**Table 3:** Neutralizing activity of RSV mAbs and RSVpreF polyclonal immune sera against RSV A and RSV B MARMs

Parental strain	Antibody resistance	Amino acid substitution	Corresponding mAb		RSVpreF immune sera	
			IC_50_ (ng/mL)	Fold resistance to parental strain	GMT (95% CI)	GMR (95% CI) to parental strain
RSV A—M37	Nirsevimab		5.8		41,552 (27,306, 63,230)	
		K68E	29.0	5	22,586 (16,069, 31,746)	0.54 (0.46, 0.65)
		N208Y	473.0	82	30,123 (20,149, 45,034)	0.72 (0.58, 0.90)
		N67I + N208Y	15,506	2,673	26,648 (19,147, 37,086)	0.64 (0.52, 0.80)
	Palivizumab		78.8		41,552 (27,306, 63,230)	
		K272E	>50,000,000^a^	>634,518	38,714 (27,169, 55,164)	0.93 (0.75, 1.15)
		K272Q	5,417,757	68,753	49,079 (33,541, 71,815)	1.18 (0.95, 1.47)
	Clesrovimab		1.5		41,552 (27,306, 63,230)	
		S443P	426.7	284	62,482 (42,043, 92,857)	1.50 (1.19, 1.90)
		S443P + K445N	382,427.0	254,951	39,622 (27,828, 56,415)	0.95 (0.78, 1.16)
		S443P + G446E	>880,000^b^	>586,667	37,568 (26,408, 53,445)	0.90 (0.74, 1.10)
RSV B—B18537	Nirsevimab		68.9		33,261 (22,667, 48,807)	
		S62R	689,133	10,002	20,221 (13,555, 30,165)	0.61 (0.47, 0.79)
		I64T	27,507	399	17,115 (11,470, 25,538)	0.51 (0.45, 0.59)
		K68E	262,304	3,807	102,866 (66,923, 158,112)	3.09 (2.47, 3.87)
		K68N	10,680	155	39,576 (27,365, 57,237)	1.19 (1.00, 1.41)
		K68Q	1,240,786	18,009	59,119 (40,816, 85,628)	1.78 (1.59, 1.99)
		N201T	296,842	4,308	41,410 (26,994, 63,525)	1.25 (0.99, 1.56)
		L203V	115,127	1,671	32,635 (20,730, 51,376)	0.98 (0.80, 1.21)
		L204I	16,653	242	45,763 (31,929, 65,593)	1.38 (1.11, 1.70)
		N208D	21,115,461	306,465	30,034 (18,903, 47,719)	0.90 (0.74, 1.11)
		N208S	815,068	11,830	58,426 (41,109, 83,038)	1.76 (1.52, 2.02)
		K68N + N201S	1,137,071	16,503	62,872 (38,200, 103,478)	1.89 (1.33, 2.69)
		K68N + N208S	16,069,197	233,225	172,444 (111,425, 266,876)	5.19 (3.79, 7.09)
	Palivizumab		71.1		33,261 (22,667, 48,807)	
		K272N	3,592,603	50,529	74,748 (49,929, 111,905)	2.25 (2.05, 2.46)
		K272Q	10,000,753	140,658	75,725 (52,043, 110,184)	2.28 (2.02, 2.57)
	Clesrovimab		2.3		33,261 (22,667, 48,807)	
		S443P	3,949.6	1,717	34,863 (23,266, 52,240)	1.05 (0.89, 1.24)
B—PFERSV277	Nirsevimab		5.1		9,099 (5,966, 13,877)	
		K65E	1,036	203	8,427 (5,640, 12,592)	0.93 (0.82, 1.05)
		K68I	380.8	75	16,320 (10,739, 24,800)	1.79 (1.38, 2.33)

*GMT* geometric mean titer, *IC*_50_ half-maximal inhibitory concentration, *mAb* monoclonal antibody, *MARM* monoclonal antibody resistant mutant, *RSV* respiratory syncytial virus.

^a^ Highest concentration tested for palivizumab and nirsevimab was 50,000,000 ng/mL.

^b^ Highest concentration tested for clesrovimab was 880 ng/mL.

In contrast to the RSV mAbs, the fold ratios in vaccine-elicited GMTs of RSVpreF immune sera remained close to or slightly above 1 (Fig. 4A–C). The GMT fold ratios for RSV A MARMs ranged from 0.54 to 1.5; RSV B MARM fold ratios were in a slightly higher range: 0.51-5.19 for all three mAb-specific resistant strains (Table 3). Overall, the data showed that RSVpreF vaccination induced robust functional neutralizing antibodies against highly resistant RSV MARMs, as well as against strains harboring naturally occurring site II and IV aa substitutions.

**Fig. 5:**
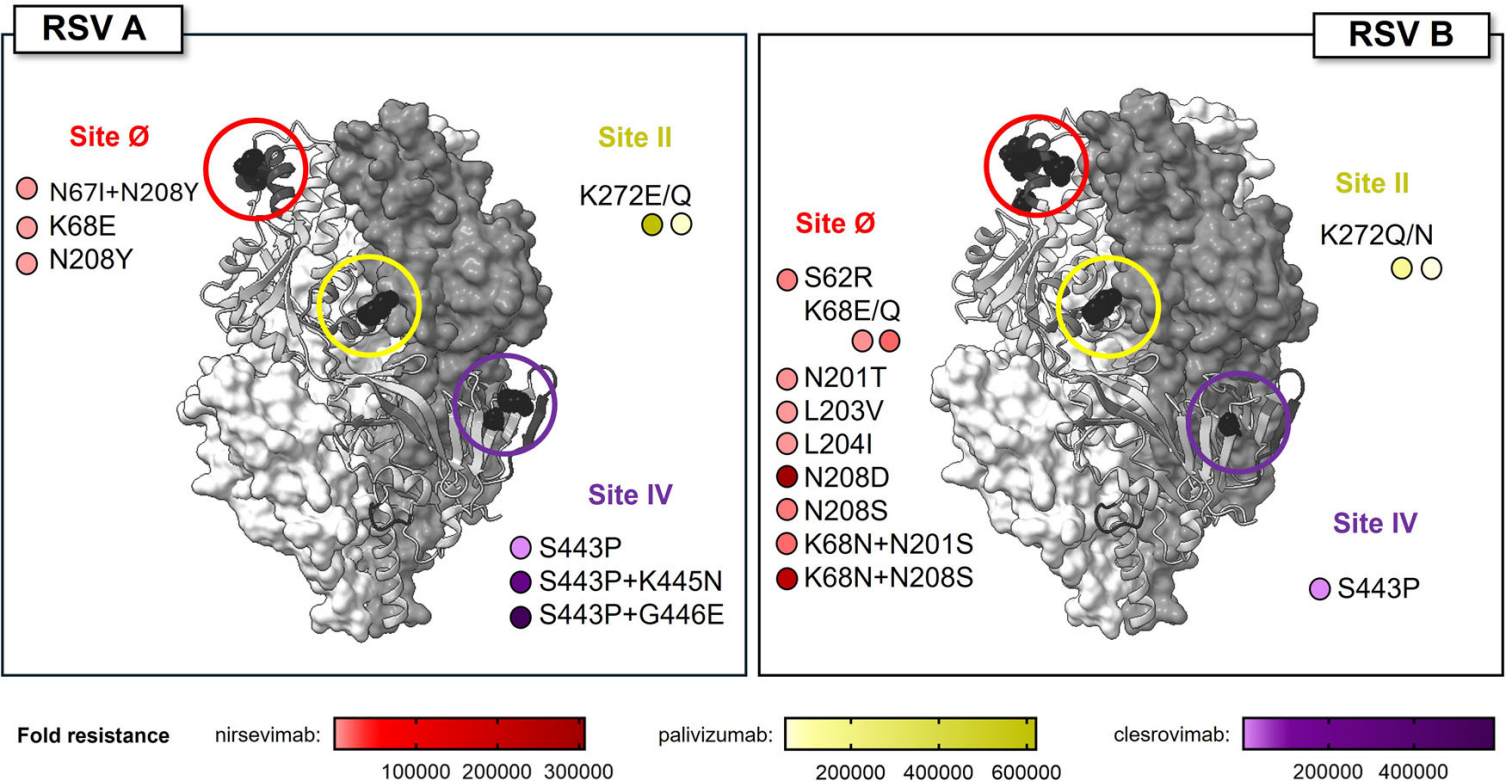
Location of the 10 amino acid residues for which variation may affect resistance to RSV preF-neutralizing monoclonal antibodies. Key amino acid (aa) substitutions or substitution combinations associated with resistance to nirsevimab, clesrovimab, and palivizumab are highlighted on the RSV A and B trimeric prefusion F (preF) three-dimensional structure. MARMs harboring specific substitutions are listed in proximity to the affected F antigenic site: site Ø (red), II (yellow), and IV (purple). Color gradients depicting fold resistance are shown separately for nirsevimab (red), palivizumab (yellow), and clesrovimab (purple). The images are based on structural data for RSV A DS-Cav1 retrieved from protein database records (Protein Data Bank ID: 4MMU).

MARMs with double substitutions showed higher mAb resistance than their corresponding single substitution MARMs. The RSV A nirsevimab-resistant double substitution mutant N67I + N208Y exhibited 33-fold higher resistance than single mutant N208Y, while RSV B double mutants K68N + N201S and K68N + N208S exhibited 20- to 1,505-fold higher resistance, respectively, compared to their corresponding single substitution mutant (Fig. 4A, Table 3).The RSV A clesrovimab double mutants S443P + K445N and S443P + G446E exhibited a similar trend with 896 and >2062-fold higher resistance, respectively, compared to single mutant S443P (Fig. 4B, Table 3). RSV A MARMs harboring S443P + G446E and S443P + K445N and RSV B MARMs harboring K68N + N208S and N208D demonstrated the highest resistance to the respective mAb. Positions of these aa substitutions are highlighted in Fig. 5 on the RSV A and B trimeric preF structure. The palivizumab-resistant substitutions contained single aa substitutions at position 272 (Fig. 4C), with the K272E mutant displaying the highest resistance as compared to K272N/Q (Table 3).

## Discussion

Both RSV and influenza are common seasonal respiratory viruses; however, unlike influenza, RSV does not undergo antigenic shifts^56^. Though the sequence of RSV F is highly conserved between the RSV A and B subgroups, antigenic differences reside in important prefusion-specific sites, and variations in surface-exposed antigenic sites are known to occur both naturally^50,54,55,57^ and induced following in vitro exposure to prophylactic RSV mAbs^40,46,53^. Here, we report for the first time the in vitro neutralization activity of RSVpreF polyclonal immune sera against a large, geographically diverse, historical and contemporary collection of RSV strains. Our findings support that the RSVpreF vaccine induces comprehensive broad-spectrum coverage across both RSV subgroups. Adult sera collected one month after RSVpreF vaccination elicited viral neutralizing antibody responses against all tested strains, including a panel of 65 contemporary RSV clinical isolates and 25 MARMs carrying aa substitutions across the six RSV F antigenic sites (Ø, I, II, III, IV, and V).

We observed that RSVpreF immune sera effectively neutralized mutants and isolates at levels comparable to the reference strains (M37 and B18537). Consistent with others^47^, we observed that L172Q and S173L, two predominant site V aa changes^58^ that contributed to suptavumab’s loss of neutralization activity against RSV B^44^, did not impact susceptibility to RSVpreF vaccine-elicited neutralizing antibody responses. We also evaluated additional key antigenic site substitutions beyond those represented in Sacconnay et al. ^47^. We identified three RSV A and two RSV B isolates with S276N/I substitutions. Changes at aa position 276 (site II) have been associated with partial resistance against palivizumab^59^ and S276 variants were recently identified in strains of both subgroups circulating in the US^35^. Isolates from Spain (*n* = 6) and South Africa (*n* = 1) collected during post-2020 RSV seasons exhibited additional site Ø and I substitutions, S211N and S389P, respectively. Apart from S276N/I, these substitutions currently have no known effect on neutralization by nirsevimab, palivizumab, or clesrovimab. Given previous observations of escape mutations in epitopes targeted by RSV F mAbs^44,59^, and as more mAb infant prophylaxes enter the RSV prevention market^16^, ongoing surveillance of potential MARMs will continue to be important over the coming years.

We previously reported sequencing analysis of RSV cases for vaccine and placebo groups from the MATISSE pivotal efficacy trial of RSVpreF in pregnant women^12^. While no antigenic site Ø mutation was observed in the RSV A cases, the vast majority ( > 80%) of RSV B sequences in the placebo and RSVpreF groups contained Ø I206M/Q209R/S211N. In the present study, the I206M + Q209R combination was present in five (38%) RSV B clinical isolates from pre-2020 RSV seasons and ten (100%) post-2020 RSV B clinical isolates. Additionally, S389P, which appeared after 2020^60^, was present in the majority ( > 65%) of RSV B cases for both groups in the MATISSE study^12^. Similarly, we found S389P in 70% of post-2020 RSV B clinical isolates. The similar frequency of RSV B cases containing I206M/Q209R/S211N or S389P between vaccine and placebo groups, together with data from this in vitro study showing effective neutralization of RSV B strains with this F phenotype, suggests that these variations are stably circulating and are not indicative of vaccine immune escape at present. A recent study^61^ placed I206M + Q209R frequency in the 2022 SH winter season at >95%; S389P frequency was reported to be 92% as recent as 2023^62^. In RSV B, monophyletic clusters arose in distinct geographic locations in the 2021/22 season due to genetic bottlenecks potentially caused by nonpharmaceutical interventions adopted against COVID-19 during the pandemic^63^. These data further substantiate that antigenic site changes, more common for the F sequence of RSV B, to date, have minimal impact on the neutralization efficiency of RSVpreF despite global prevalence. Sequence variations being more common in RSV subgroup B than subgroup A have been consistently reported across studies^34–37^, though the molecular mechanisms behind this observation remain unclear.

Several RSV F aa substitutions in sites Ø and V are reported to impair or abolish the activity of RSV human mAbs specific to the prefusion conformation. K68N/Q and N201S/T in site Ø, for example, were shown to dramatically reduce the susceptibility of the virus to in vitro neutralization by nirsevimab^57^. In our study, the nirsevimab MARMs containing N67I + N208Y for RSV A, and K68N + N208S and N208D for RSV B, exhibited the highest level of resistance to nirsevimab, consistent with Zhu et al. ^46^. During the 2023/24 RSV season in France, Fourati et al.^54^ identified N208D and the I64M + K65E combination in two RSV B nirsevimab breakthrough infections. Available RSV genomic surveillance data during this period showed that the emergence of such strains did not precede the rollout of nirsevimab. A query of the GISAID EpiRSV database for N208D using December 31, 2023, as the collection cut-off date identified three strains containing N208D (I206M/N208D/Q209R/S211N); all were detected in isolates collected in November 2023 from Spain and France, directly following implementation of a nation-wide nirsevimab immunization campaign in those countries^64–66^. I64T, K68E, and N208S mutants have also been identified in nirsevimab-treated cases, exhibiting a 200-to-400-fold increase in resistance to nirsevimab^50,55^. All three substitutions were observed in nirsevimab MARMs in this study.

In the present study, MARMs with the most resistance to clesrovimab harbored S443P/G446E/K445N substitutions among both subgroups. This is consistent with Tang et al., who reported clesrovimab escape mutants with IC_50_ > 1 ug/mL containing G446E, S443P, S443P + K445N, or S443P + G446E (RSV A) and S443P (RSV B)^40^. MARMs with the most resistance to palivizumab contained K272E/Q/N substitutions among both subgroups. Bates et al. also identified palivizumab-induced K272E/Q substitutions in RSV-A2, and mutants containing K272E/Q were demonstrated to be highly resistant to palivizumab in vitro^53^.

The present study has limitations. Due to the rarity of naturally occurring clesrovimab binding site (site IV) substitutions^67^, our RSV A and RSV B isolate collection did not include representative natural clesrovimab-resistant strains. As a result, we were unable to assess clesrovimab neutralizing activity relative to reference strains, although we will continue to monitor changes in site IV as clesrovimab is newly deployed. Additionally, we obtained only two RSV A and one RSV B strains from South America (Chile) and none from Asia. Due to the COVID-19 pandemic and associated social distancing, we were also not able to source isolates from the 2020/21 RSV season. While neutralization abilities of RSVpreF immune sera were assessed at an early post-vaccination time point (1 month), sera from extended time points post-vaccination (e.g., at the end of the first or at the beginning of the second RSV season) were not evaluated. Although sera from pregnant individuals, infants, or older adults were not tested in this study, it has been shown that RSVpreF neutralizing responses in pregnant individuals are similarly robust compared to nonpregnant adults, and also when comparing young adults and older adults^4,9^. Nevertheless, future research should assess neutralizing potency of RSVpreF immune sera across all demographics receiving the vaccine.

In summary, our findings revealed the ability of bivalent RSVpreF to induce a broadly neutralizing antibody response against RSV A and RSV B strains from multiple seasons and countries bearing antigenically diverse F protein variants. The evolutionary trajectory of RSV F post-pandemic and the emergence and spread of nonsynonymous aa substitutions in key antigenic sites, currently rarely identified in epidemiological surveillance studies, could reduce the effectiveness of existing RSV interventions, in particular prophylactic mAbs that additionally provide distinct selective pressures. Our study highlights the importance of continued surveillance of RSV F sequences with expanded use of RSV vaccines and prophylactic mAbs. The most effective RSV therapies will provide broad protection against both current and emerging strains of RSV, and our study demonstrates the value of RSVpreF vaccination in generation of a polyclonal antibody response that neutralizes current and emergent strains of RSV, along with mitigating the immune escape observed with RSV mAbs, which target variable sites on RSV F. Due to the evolving epidemiological landscape of RSV, driven by its complete absence during the height of the COVID-19 pandemic, subsequent resurgence, and the implementation of new RSV preventative measures beginning in 2022, it remains essential to maintain global surveillance efforts.

## Methods

### Reference strain and clinical isolate virus propagation

RSV reference strains A/Memphis/37/2001 (M37; Genbank accession KM360090) and B18537 (B subgroup) were propagated in HEp-2 cells (ATCC CCL-23) as previously described^68^ and titered in A549 cells (ATCC CCL-185) for use in RSV neutralization assays. Briefly, semiconfluent monolayers were infected at a multiplicity of infection (MOI) of 0.001 in SFM4MegaVir medium supplemented with sodium bicarbonate, GlutaMAX-I (100x), non-essential amino acids (100x), penicillin-streptomycin, and sodium pyruvate. Flasks were monitored for cytopathic effects (CPE) and harvested 5–6 days post infection. Clarified supernatants supplemented with 1X SPG were frozen and stored at −80 °C. Virus titers (FFU/mL) were determined by RSV infectivity assay (see below). Clinical RSV isolates were sourced from the United States, Chile, Netherlands, and South Africa during 2015 through 2019 (pre-COVID-19 pandemic), or from the US, South Africa, and Spain during 2021 through 2023. Virus stocks were propagated from original nasal specimens and passaged 1-3 times in HEp-2 cells as described above to generate sufficient virus for serum neutralization testing.

### Whole genome sequencing analysis of RSV strains

WGS of the clinical isolates and MARMs are as previously described^69,70^. In brief, viral RNA was extracted using QIAamp Viral RNA Mini Kit (Qiagen, Germantown, MD) according to the manufacturer’s protocol followed by DNase treatment and RNeasy MinElute (Qiagen) clean up. Next, Ovation RNA-Seq System V2 (Tecan, Morrisville, NC) was used to synthesize complementary DNA from the purified viral RNA. The sequencing library was prepared using the Nextera XT (Illumina, San Diego, CA) followed by loading onto a MiSeq instrument using a MiSeq 2×300 cartridge. For genomic sequence analysis, raw reads were aligned to reference genomes of RSV A isolate RSVA/Homo sapiens/USA/LA2_21/2013 (Genbank accession KJ672443) or RSV B strain RSVB/Homo sapiens/PER/FPP00592/2011 (Genbank accession KJ627247.1) using the “Map Reads to Reference” tool in CLC Genomics Workbench v21.0.5 (Qiagen) with default parameters. Consensus sequences were extracted using the “extract consensus sequence” tool with a cut-off 30x sequencing coverage, and conflict nucleotides were resolved by vote. Amino acid changes in the viral genome were identified using the “Basic Variant Detection” program with the cut-off for SNP set at >50% sequence heterogeneity and at least 30x sequencing coverage.

### RSVpreF immune sera

Human sera were from healthy, RSV-seropositive Phase 1/2 trial participants (*n* = 30), 18–49 years of age (NCT03529773), who were vaccinated intramuscularly with a single 120 µg dose of RSVpreF without Al(OH)_3_ (Abrysvo® formulation). Participant sera utilized in this study, collected prior and one month post vaccination^4^, were tested against a panel of RSV clinical isolates and MARMs (see sections below).

### Anti-RSV F monoclonal antibodies

The palivizumab used in this study was a commercial product (Synagis®; 66658-231-01) purchased from Clinigen (Yardley, PA). Nirsevimab (MEDI8897)^71^ and clesrovimab (Rb1)^40^ were generated in-house with the same heavy and light chain variant regions as the original mAbs, as previously described^40,71^. Both MEDI-8897 and Rb1 were constructed as human IgG1 without the YTE half-life extending mutation at FC region and purified from supernatant of transiently transfected expiCHO (MEDI-8897) or Expi293F cells (Rb1). Purification of MEDI-8897 was performed by MabSelect SuRe affinity chromatography, followed by dialysis into phosphate-buffered saline (PBS), pH 7.4, and 0.2 µm filtration. Rb1 was purified with Protein A magnetic beads per the manufacturer’s protocol. Quality of the purified mAbs was assessed by SDS-PAGE. IgG concentration was determined by measuring UV absorbance at 280 nm using a NanoDrop instrument (ThermoFisher Scientific). In-house MEDI-8897 was used to generate all nirsevimab-resistant viral strains, while neutralization activity of commercial nirsevimab (Beyfortus®; 49281-574-15, Clinigen) was assessed in all assays utilizing clinical isolates or nirsevimab-resistant viral strains, except PFERSV277 and respective mutants.

### Generation of RSV monoclonal antibody-resistant mutant strains (MARMs)

RSV A and B strains resistant to nirsevimab, palivizumab, and clesrovimab were generated by growing virus in the presence of the individual mAb (Fig. S6A)^46,72^ or using a standard reverse genetic approach (Fig. S6B)^73,74^. For MARMS generated via serial culture in the presence of mAb, RSV reference strain B18537 and clinical isolate PFERSV277 (B subgroup) at MOI of 0.001–0.3 were incubated with 10 times IC_50_ concentration of in-house generated nirsevimab at 37 °C for one hour. HEp-2 cell monolayers in T25 flasks were inoculated with the virus/mAb mixture and incubated at 37 °C for 5–7 days until CPE reaching 75% or higher. Viruses were titrated and used for the next round of infection. The procedure was repeated two more times (*n* = 3 passages). The individual viruses were isolated by limiting dilution in 96-well plates and propagated in HEp-2 cells without mAb to establish virus stocks of MARMs.

For MARMS generated recombinantly based on literature-reported escape mutations, RSV entry vectors (pCC1FOS vector backbone (NovoPro, Cat# V008674)) were constructed encoding the antigenome of A/Memphis/37/2001 (M37) or B/B9617 strains with the F gene omitted and replaced by a spacer flanked by *BsaI* restriction enzyme sites, adapted from Hotard et al. ^73^. The 3’ leader sequence was preceded by a T7 promoter, and the 5’ trailer sequence was flanked by sequences encoding the hepatitis D virus ribozyme and the polyadenylation signal of bovine growth hormone. Separate subcloning plasmids encoding the native F gene from either M37 or B18537 strains, flanked by *BsaI* sites, were constructed via site-directed mutagenesis. Golden Gate assembly via *BsaI* sites (NEB) was completed with the pFOS_RSV entry vector and a F subcloning plasmid to generate final bacmid construct encoding full-length cDNA antigenomes (cRSV). Additional helper plasmids were constructed encoding codon-optimized M37 N, P, M2-1, and L proteins cloned into a pcDNA3.1(+) mammalian expression vector.

RSV recombinant MARMs were rescued in BSR-T7 cells (a BHK-21 derivative constitutively expressing T7 polymerase) as described previously^75^. In brief, the overnight monolayer of BSR-T7 was transfected with cRSV (1 µg) and helper plasmids N, P, M2-1 (0.5 µg of each), and L (0.25 µg) using Lipofectamine 3000 (Invitrogen) according to manufacturer’s instructions in triplicate in a 6-well plate. Additional OptiMEM was added at 4 h post-transfection. At 16 h post-transfection, media were changed to Dulbecco’s Modified Eagle Medium (DMEM) supplemented with 3% fetal bovine serum (FBS). Every 2–4 days, cells were disassociated or mechanically scraped and scaled up into a 25 cm^2^ and further to 175 cm^2^ tissue-culture flask. At days 10-11, 1/6th of cell/supernatant suspension from T175 flask was transferred to a new 175 cm^2^ flask with additional fresh medium. Flasks were incubated for an additional 3-4 days until CPE was observed, at which point virus was harvested and denoted as P0.

Rescued recombinant MARMs were amplified 1-3 passages in HEp-2 cells and F protein amino acid sequences were confirmed by WGS.

### RSV neutralization and infectivity assays

Two types of RSV neutralization assays were utilized as follows: 1) a validated clinical assay (384-well microplate) and 2) an RSV non-clinical neutralization assay (96-well microplate). The former is based on the non-clinical neutralization assay. In both neutralization assay formats, the same RSV viral strains (M37, A subgroup; or B18537, B subgroup), cell line (A549), and the same primary antibody (pre-F monoclonal antibody, Pfizer: N50-9) were used for the detection of focus-forming units.

For the clinical neutralization assay, serum neutralizing levels of antibodies to RSV A and RSV B were evaluated in separate RSV A and RSV B neutralization assays, as described previously^4^. Briefly, nirsevimab was spiked into pooled human sera representative of 5 non-vaccinated donors. A baseline neutralizing activity of the human serum diluent (due to prior RSV infection) was calculated and subtracted from the results. Three independent preparations were created for each assay and assessed over 0X, 0.5X (3.4 µg/mL), 1.0X (10.2 µg/mL), and 2.0X (13.6 µg/mL) fold dilutions over the 90% effective concentration threshold of 6.8 µg/mL identified in infants^52^. Neutralization titers were calculated as the interpolated reciprocal of the serum dilution resulting in a 50% reduction in the number of viral focus-forming units when compared with the control wells without serum.

The RSV non-clinical neutralization assay, a research assay described previously^33^ with similar methodology as the qualified/validated clinical RSV neutralization assay, was used with minor modifications. Briefly, serial dilutions (3-fold) of heat-inactivated human immune sera or purified mAb were mixed with clarified cell culture medium containing RSV—either strain M37 (A subgroup) or B18537 (B subgroup)—for 1 hour and transferred to an A549 (ATCC CCL-185) cell monolayer in a 96-well plate. The mixture containing the cells, virus and serum was incubated for 22–24 h, and focus-forming units were revealed by incubation with a primary anti-RSV F mAb (N50-9; Pfizer Inc., Pearl River, NY) followed by a secondary Alexa488-conjugated antibody (Invitrogen, A-11001). Fluorescently labeled viral foci were enumerated by a CTL Immunospot Analyzer (Cellular Technology Limited). For human immune sera, the data were expressed as NT_50_ that were calculated as the reciprocal serum dilutions at which 50% of the virus was neutralized compared to control wells without serum. Sera that failed to neutralize at the lowest serum dilution of 1:20 were reported to have a neutralizing titer of 20 (LLOD) For the mAbs, the data were expressed as half maximal inhibitory concentration (IC_50_), calculated as the concentration of the mAb required to neutralize 50% of the virus as compared to control wells without the mAb.

The RSV infectivity assay is similar in principle to the neutralization assays. Serial dilutions of virus were inoculated onto A549 cell monolayers in 96-well plates. At the end of a 22–24 h incubation, immunostaining and enumeration of viral foci were performed following the same protocol above. Viral titer (FFU/mL) was determined by calculating the foci per well multiplied by the dilution factor and volume of inoculum.

### Statistical analysis

All statistical analyses were performed by an ANOVA on log-transformed data using SAS version 9.4. Appropriate multiple comparisons tests were performed to control the significance level at 0.05 within each RSV strain. The 50% neutralizing GMTs, GMR relative to the reference or parental strains, and 95% confidence intervals were analyzed using SAS version 9.4.

## Supplementary information

Supplementary Materials

## Data Availability

Primary nucleotide sequence data for the RSV A and RSV B clinical isolates analyzed in this study have been deposited to the NCBI SRA database under BioProject Accession PRJNA1308556. Materials or cell line requests should be submitted to the corresponding author and may be subject to approval by Pfizer or the vendor who generated the material/cells on behalf of Pfizer. All other data needed to evaluate the conclusions in the paper are present in the paper and/or the Supplementary Materials.
