# Supplementary material for: Bivalent RSV prefusion F vaccination elicits effective neutralization of contemporary and monoclonal antibody-resistant RSV strains
Source: NPJ Vaccines. 2026 Mar 14;11:93. doi: 10.1038/s41541-026-01418-8 (PMC13133364; doi:10.1038/s41541-026-01418-8)
Supplement: Supplementary file 1 — Supplementary Materials [file 41541_2026_1418_MOESM1_ESM.pdf]

Supplementary Materials for

Bivalent RSV Prefusion F Vaccination Elicits Effective Neutralization of Contemporary and Monoclonal Antibody-Resistant RSV Strains

Wei Chen, Lyndsey Martinez, Larissa Falcao, Zhenghui Li, Chaitanya Kurhade, Helene Boigard, Vidia Roopchand, Imani Richardson, Trisha Dasgupta, Katrina Llamera, Jing Colatat, Linda Goding Brock, Annaliesa S. Anderson, Kena A. Swanson*

Contents

Supplementary Figure 1. WGS workflow for the selection of the RSV A and B clinical isolate panel 2

Supplementary Figure 2. Country of collection and RSV seasons represented in the RSV clinical isolate panel. 4

Supplementary Figure 3. F protein sequence diversity of RSV clinical isolate panel. 4

Supplementary Figure 4. RSVpreF immune sera effectively neutralizes globally circulating clinical RSV A and RSV B strains from the 2015 through 2022. 5

Supplementary Figure 5. Susceptibility of RSV A and RSV B reference strains to nirsevimab above and below the established nirsevimab protective concentration of 6.8 µg/mL. 6

Supplementary Figure 6. Strategies for Generation of Monoclonal Antibody Resistant Mutants (MARMs) of RSV A and RSV B 7

Supplementary Table 1. Global prevalence of RSV A and B genome sequences bearing F protein amino acid (AA) substitutions in antigenic sites Ø, I, II, III, and V. 8

Supplementary Table 2. Neutralizing GMTs of RSVpreF sera against RSV A clinical isolates. .. 9

Supplementary Table 3. Neutralizing GMTs of RSVpreF sera against RSV B clinical isolates. .11

Supplementary Table 4. IC_{50} of nirsevimab against select RSV A and B clinical isolates. 12

Supplementary Table 5. Global prevalence of RSV B genome sequences containing triple I206M/Q209R/S211N, double I206M/Q209R, and single S211N F protein amino acid substitutions in GISAID, 2018-2024. 13

References 14

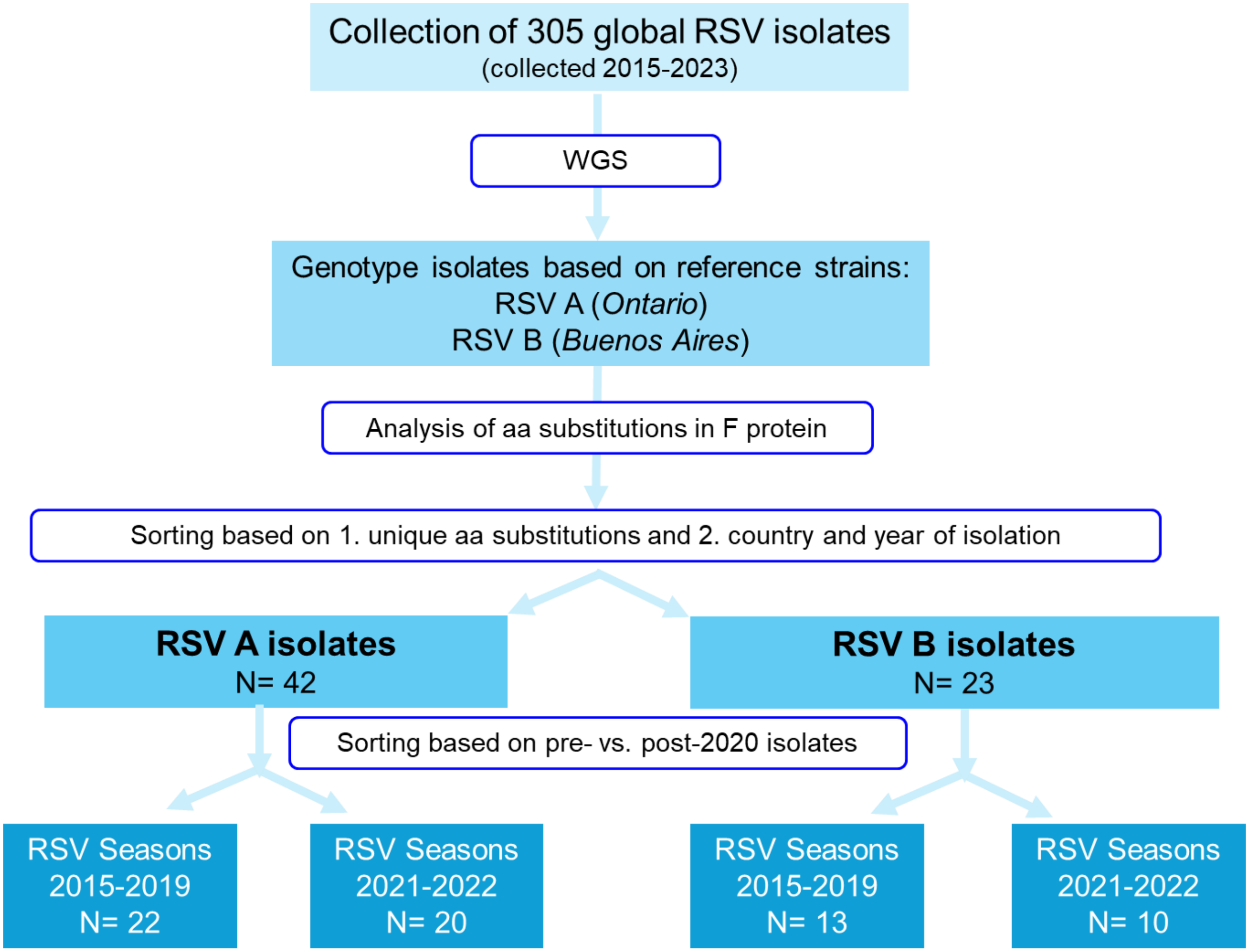

Supplementary Figure 1. WGS workflow for the selection of the RSV A and B clinical isolate panel. From an internal collection of 305 contemporary clinical RSV isolates, collected from 2015 through 2023, RSV A and B were confirmed to be Ontario and Buenos Aires genotypes, respectively, by whole genome sequencing (WGS), based on reference strains RSVA/Homo sapiens/USA/LA2_21/2013 (RSV A) and RSVB/Homo sapiens/PER/FPP00592/2011 (RSV B). WGS analysis was further used to identify genotyped isolates in the collection bearing amino acid (aa) polymorphisms in the full-length F protein, relative to the bivalent RSVpreF vaccine antigen sequences. Sorting for the final panel was based on 1) unique aa substitutions and 2) country and year of isolation. From this, 65 isolates were selected: 42 RSV A isolates and 23 RSV B isolates. Isolates were then sorted based on pre- or post-2020 isolation (the 2020-2021 RSV season was not represented in our collection). Pre-2020 isolates consisted of 22 and 13, RSV A and B isolates, respectively. Post-2020 isolates consisted of 20 and 10 RSV A and B isolates, respectively.

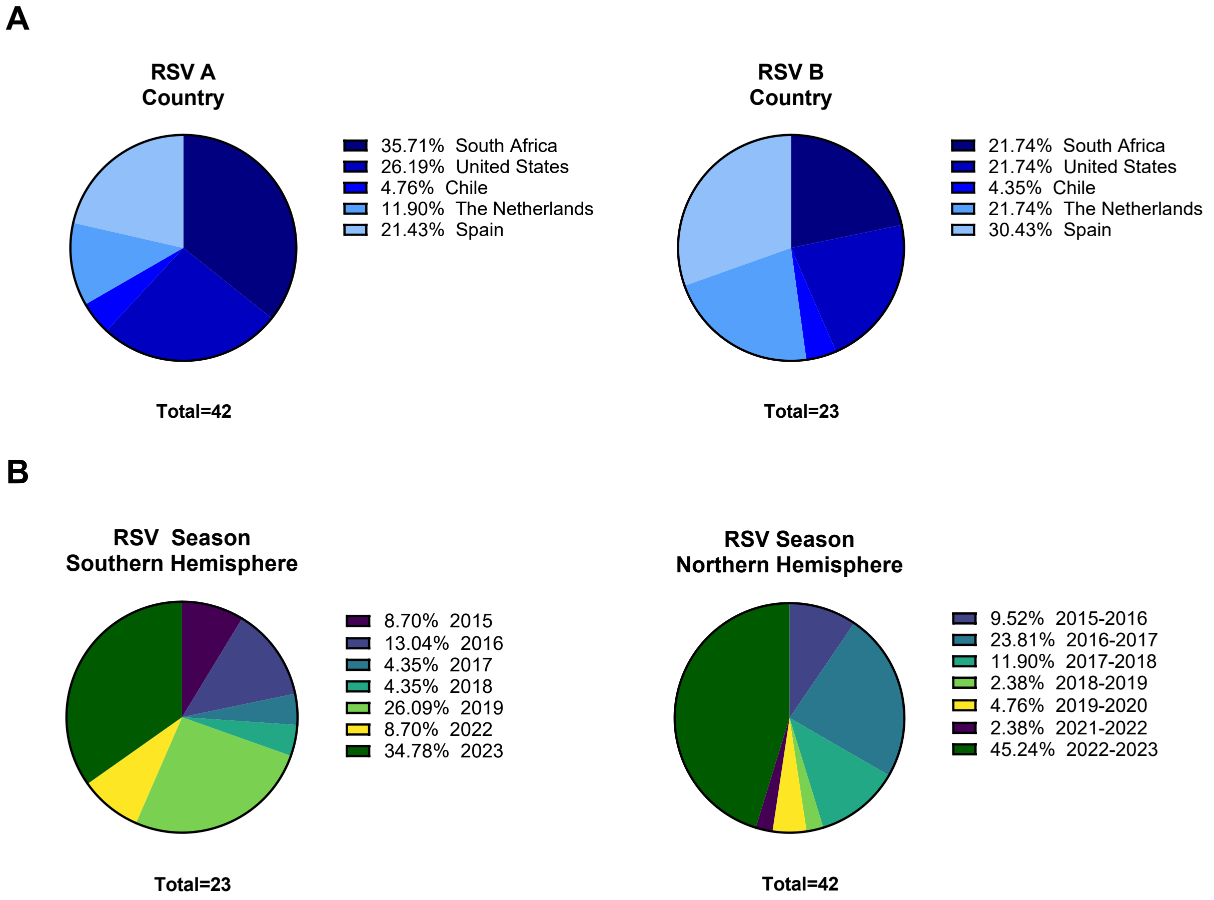

Supplementary Figure 2. Country of collection and RSV seasons represented in the RSV clinical isolate panel. Information on the (**A**) country of collection and (**B**) RSV season of the 65 RSV clinical isolates. A. Isolates were collected from five countries: South Africa, the United States, Chile, The Netherlands, and Spain. B. Isolates were from seven southern hemisphere RSV seasons (2015, 2016, 2017, 2018, 2019, 2022, 2023) and seven northern hemisphere RSV seasons (2015/16, 2016/17, 2017/18, 2018/19, 2019/20, 2021/22, 2022/23). The 2020/21 RSV season was not represented in our collection due to low circulation of RSV and other respiratory viruses during the original wave of the COVID-19 pandemic.

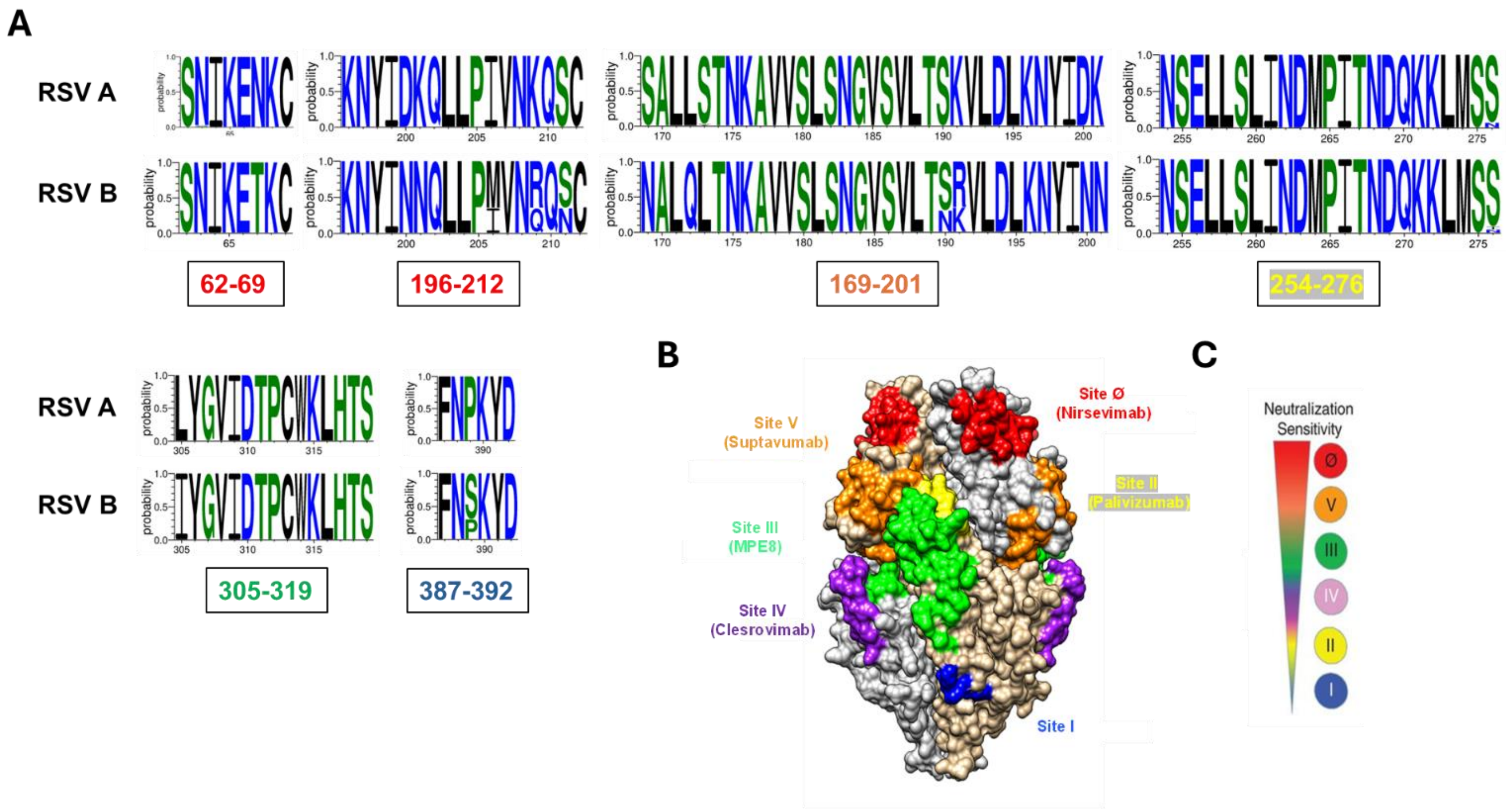

Supplementary Figure 3. F protein sequence diversity of RSV clinical isolate panel. Sequence logo of the 65 RSV A and RSV B clinical isolates based on the F amino acid sequence alignment of antigenic sites Ø, I, II, III, and V. **A.** The sequence logo is arranged as site Ø (62-69, 196-212), site V (169-201), site **II** (254-276), site **III** (305-319) and site **I** (387-392). **B.** Colored amino acid position numbers shown in (**A**) correspond to the antigenic epitopes highlighted in the RSV pre-fusion F (preF) structure. **C.** Neutralization sensitivity hierarchy of the antigenic sites shown in (**B**), adapted from Graham 2019 [1].

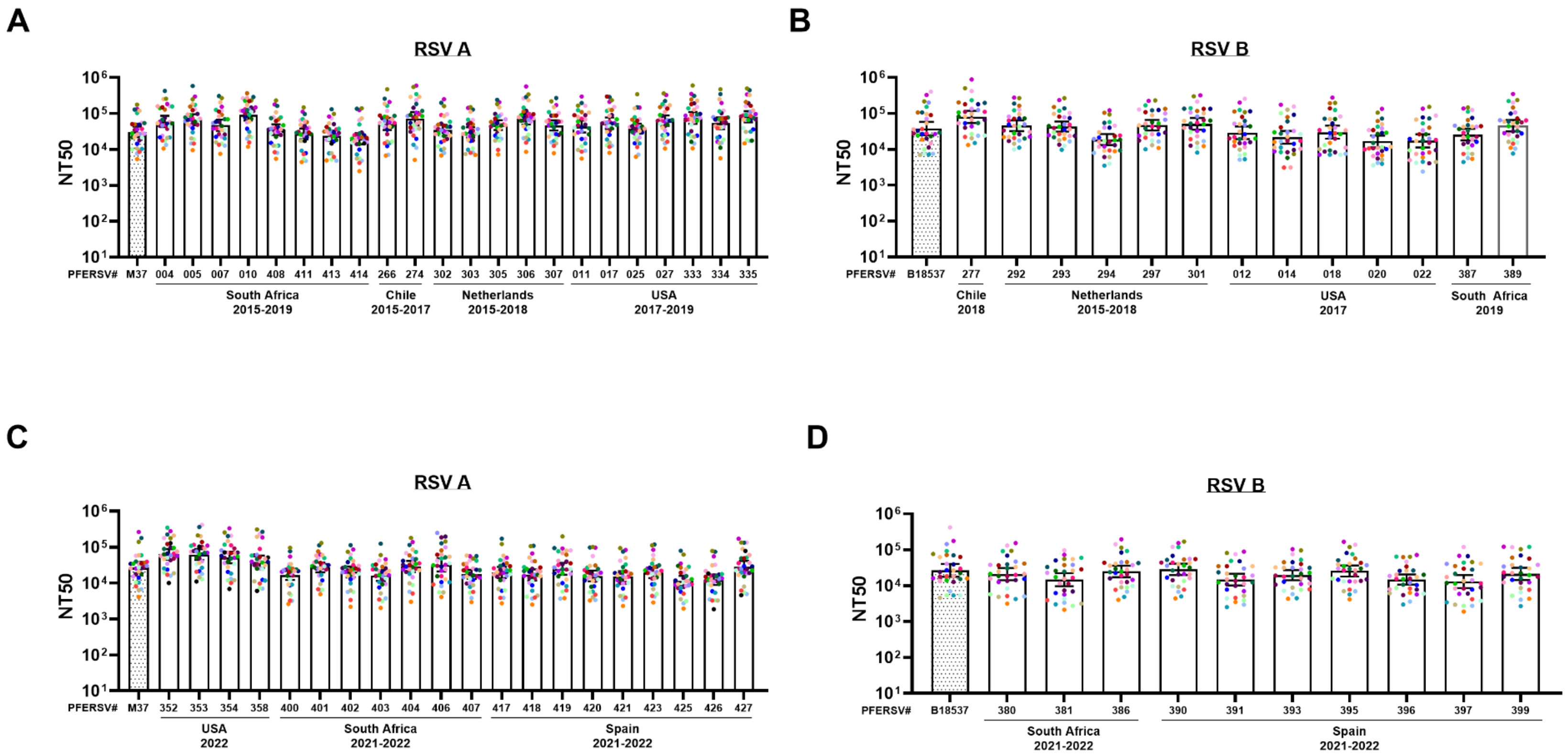

Supplementary Figure 4. RSVpreF immune sera effectively neutralizes globally circulating clinical RSV A and RSV B strains from 2015 through 2022. Immune sera collected one month after trial participants were vaccinated with a single dose of RSVpreF (NCT03529773) (n = 30) were tested against a panel of (**A**) pre-2020 (n=22) (**C**) 2021-2022 RSV A clinical isolates (n=20), as well as (**B**) pre-2020 (n=13) and (**D**) 2021-2022 RSV B clinical isolates (n=10). Reference strains M37 and B18537 in the RSV neutralization assay (patterned bars) are shown for RSV A (A and B) and RSV B (C and D), respectively. The 50% virus neutralizing titers (NT_{50}) of individual participant sera are represented by colored dots. The NT_{50} geometric mean titer (GMT) per strain or isolate is represented by bars.

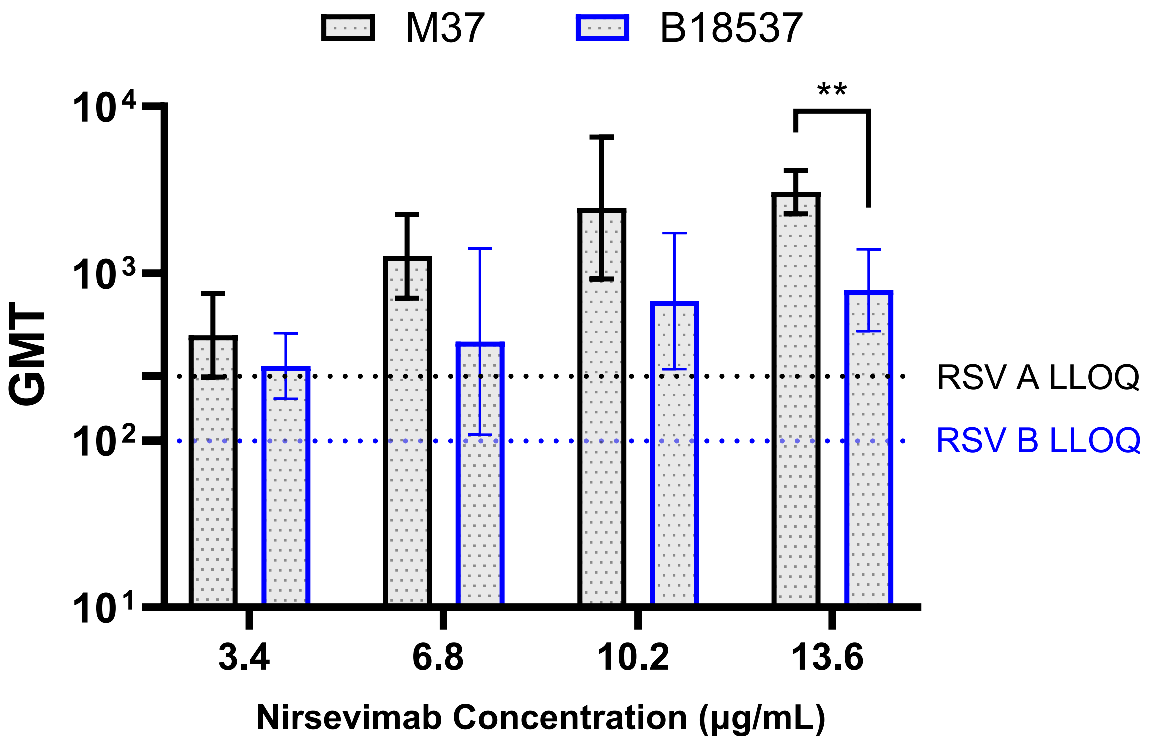

Supplementary Figure 5. Susceptibility of RSV A and RSV B reference strains to nirsevimab above and below the established nirsevimab protective concentration of 6.8 µg/mL. Commercial nirsevimab (Beyfortus) was diluted to the indicated concentrations (3.4, 6.8, 10.2 and 13.6 ug/mL) in the pooled sera of five non-RSVpreF-vaccinated adult human donors, and the samples were tested in each validated RSV neutralization assay for neutralization activity against reference strains M37 (RSV A) and B18537 (RSV B). 50% neutralizing titers are reported with subtraction of the baseline neutralization per assay plate, which is due to prior RSV infection. The bars represent the geometric mean titer (GMT) of n=60+ replicates collected per concentration evaluated, with 95% CI (error bars). The black and blue dashed lines represent the lower limit of quantitation (LLOQ) for RSV A (y=242) and B (y=99). Statistical significance was determined by ANOVA and adjusted using a Bonferroni multiple comparisons test. **p<0.01.

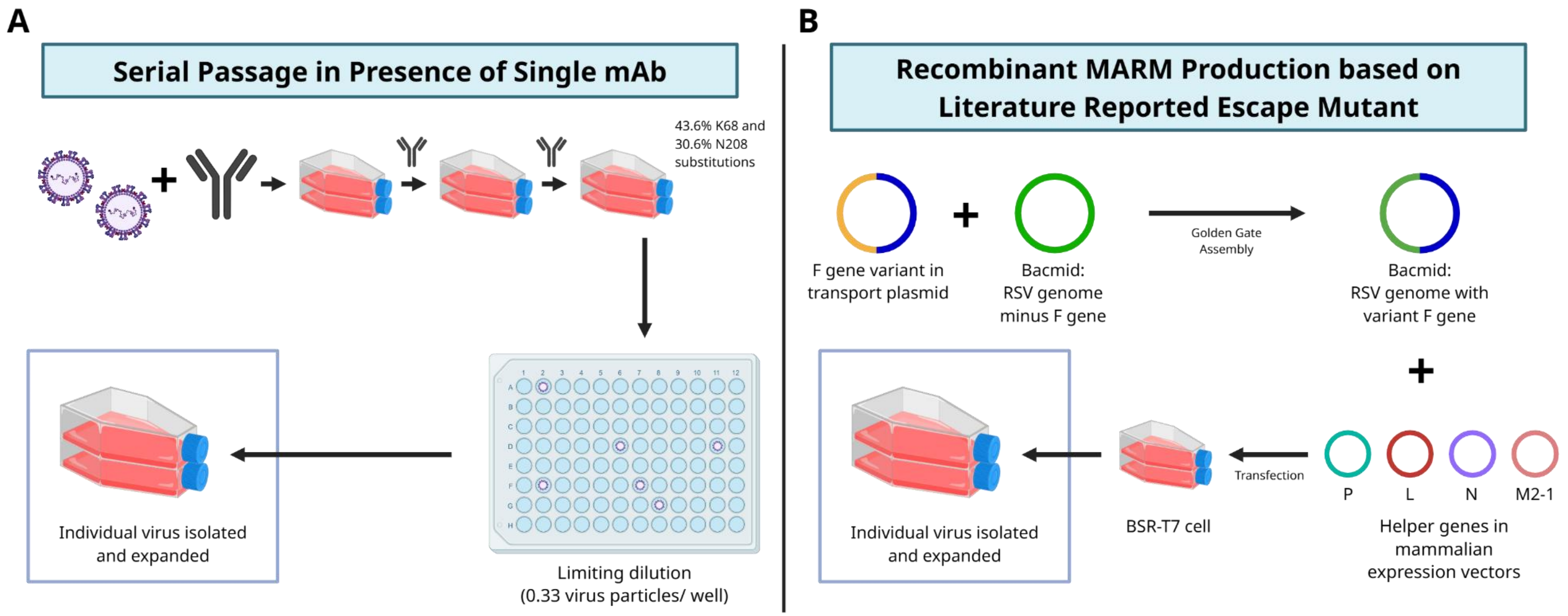

Supplementary Figure 6. Strategies for Generation of Monoclonal Antibody Resistant Mutants (MARMs) of RSV A and RSV B. RSV A and B MARMs were generated by either (A) propagating the virus under mAb pressure or (B) using a reverse genetics approach. **A.** RSV A and B reference and clinical isolate strains at MOI of 0.001-0.3 were incubated with 10 times IC50 concentration of the mAb and transferred to HEp-2 cell monolayers. Flasks were monitored for cytopathic effect and the supernatants were harvested for each subsequent round (n=3 passages) of infection in the presence of the mAb. The individual virus populations were isolated by limiting dilution, expanded and the MARMs were identified by WGS. **B.** Bacmids encoding the antigenome of A/Memphis/37/2001 (M37) and B/B9617 strains were constructed as RSV entry vectors. Separate subcloning plasmids encoding the native F gene from either M37 or B18537 strains, were constructed and F antigenic site mutations were introduced via site-directed mutagenesis. Golden Gate assembly was used with the RSV entry vector and the F subcloning plasmid to generate final bacmid construct encoding full-length cDNA antigenomes (cRSV). The bacmid along with helper plasmids, M37 N, P, M2-1, and L proteins in a pcDNA3.1(+) mammalian expression vector were used to transfect BSR-T7 cells and MARMs were isolated and expanded. Created in BioRender. Martinez, L. (2026) https://BioRender.com/vi88oo3

Supplementary Table 1. Global prevalence of RSV A and B genome sequences bearing F protein amino acid (AA) substitutions in antigenic sites Ø, I, II, III, and V.

**RSV A**	**AA substitution**	**Incidence**[a]	**Frequency (%)**[b]
**Site Ø**	N63S	136	0.39
	A74T	56	0.16
**Site II**	S276N	1824	5.21
**Site V**	S173Y	0	0.00
	S173L+S173T=27+3	30	0.09
**RSV B**	**AA substitution combinations**	**Incidence**[a]	**Frequency (%)**[c]
	F45L (Site III), L172Q (Site V), S173L(Site V)	7733	31.38
	F45L(Site III), L172Q(Site V), S173L(Site V), I206M (Site 0), Q209R (Site Ø)	5459	22.15
	F45L(Site III), L172Q(Site V), S173L(Site V), I206M (Site Ø), Q209R (Site Ø), S276N (Site II),	347	1.41
	F45L(Site III), L172Q(Site V), S173L(Site V), I206M (Site Ø), Q209R (Site Ø), S211N(Site Ø), S389P (Site I)	361	1.46
	F45L(Site III), L172Q(Site V), S173L(Site V), I206M (Site Ø), Q209R (Site Ø), S211N(Site Ø), S276I (Site II), S389P (Site I)	0	0.00
	F45L(Site III), L172Q(Site V), S173L(Site V), I206M (Site Ø), Q209R (Site Ø), S211N(Site Ø)	581	2.36

[a] GISAID query results used December 31, 2024 as the cut-off date

[b] RSV A total count, n= 35,001

[c] RSV B total count, n=24,643

Supplementary Table 2. Neutralizing GMTs of RSVpreF sera against RSV A clinical isolates.

Clinical isolate ID	Sera samples (n)	GMT (95% CI)	GMR relative to reference strain (95% CI)
M37 (Ref)	30	30,976 (22,105, 43,407)	1.00
PFERSV004	30	60,287 (41,956, 86,629)	1.95 (1.66, 2.28)
PFERSV005	30	67,350 (47,519, 95,457)	2.17 (1.94, 2.44)
PFERSV007	30	47,621 (33,463, 67,770)	1.54 (1.36, 1.73)
PFERSV010	30	90,825 (64,060, 128,773)	2.93 (2.36, 3.65)
PFERSV011	30	43,525 (29,655, 63,883)	1.41 (1.18, 1.67)
PFERSV017	30	53,915 (37,487, 77,647)	1.74 (1.50, 2.02)
PFERSV025	30	37,888 (27,216, 52,746)	1.22 (1.07, 1.39)
PFERSV027	30	63,701 (45,315, 89,547)	2.06 (1.85, 2.29)
PFERSV266	30	51,017 (34,715, 74,975)	1.65 (1.44, 1.88)
PFERSV274	30	72,229 (47,293, 110,313)	2.33 (1.88, 2.89)
PFERSV302	30	34,672 (24,733, 48,607)	1.12 (1.00, 1.25)
PFERSV303	30	29,965 (21,643, 41,488)	0.97 (0.90, 1.04)
PFERSV305	30	47,719 (34,829, 65,379)	1.54 (1.37, 1.73)
PFERSV306	30	70,625 (49,278, 101,220)	2.28 (2.02, 2.57)
PFERSV307	30	46,831 (33,685, 65,108)	1.51 (1.37, 1.67)
PFERSV333	30	75,748 (51,991, 110,359)	2.45 (2.06, 2.91)
PFERSV334	30	53,607 (36,715, 78,269)	1.73 (1.45, 2.06)
PFERSV335	30	81,792 (57,337, 116,678)	2.64 (2.20, 3.17)
PFERSV408	30	35,754 (25,378, 50,373)	1.15 (1.07, 1.25)
PFERSV411	30	27,083 (19,217, 38,169)	0.87 (0.76, 1.01)
PFERSV413	30	24,341 (17,535, 33,788)	0.79 (0.70, 0.89)
PFERSV414	30	19,853 (13,646, 28,882)	0.641 (0.59, 0.70)
M37 (Ref)	30	27,080 (19,016, 38,564)	1.00
PFERSV352*	30	62,724 (44,630, 88,155)	2.32 (2.02, 2.66)
PFERSV353*	30	61,957 (43,735, 87,771)	2.29 (1.91, 2.75)
PFERSV354*	30	51,418 (35,448, 74,583)	1.90 (1.68, 2.15)
PFERSV358*	30	38,897 (25,982, 58,233)	1.44 (1.23, 1.67)
PFERSV400*	30	16,831 (12,139, 23,336)	0.62 (0.55, 0.71)
PFERSV401*	30	27,342 (20,054, 37,280)	1.01 (0.87, 1.18)
PFERSV402*	30	20,366 (14,487, 28,631)	0.75 (0.67, 0.84)
PFERSV403*	30	16,083 (11,389, 22,711)	0.59 (0.52, 0.68)
PFERSV404*	30	28,967 (20,315, 41,304)	1.07 (0.95, 1.21)
PFERSV406*	30	32,109 (21,070, 48,933)	1.19 (0.84, 1.68)
PFERSV407*	30	17,521 (13,013, 23,592)	0.65 (0.56, 0.75)
PFERSV417*	30	19,799 (13,910, 28,183)	0.73 (0.63, 0.85)
PFERSV418*	30	16,885 (11,839, 24,080)	0.62 (0.56, 0.70)
PFERSV419*	30	25,020 (17,148, 36,506)	0.92 (0.78, 1.10)
PFERSV421*	30	15,050 (10,647, 21,273)	0.56 (0.51, 0.61)
PFERSV420*	30	15,740 (11,141, 22,238)	0.58 (0.53, 0.64)
PFERSV423*	30	19,373 (13,777, 27,241)	0.72 (0.63, 0.81)
PFERSV425*	30	11,517 (8,326, 15,932)	0.43 (0.36, 0.50)
PFERSV426*	30	12,369 (8,836, 17,314)	0.46 (0.42, 0.50)
PFERSV427*	30	28,442 (19,605, 41,262)	1.05 (0.90, 1.22)

All Isolates	33,968 (32,275, 35,749)	1.18 (1.15, 1.20)

GMT, geometric mean titer; GMR, geometric mean ratio; Ref, reference strain
*Post-2020 RSV isolates

Supplementary Table 3. Neutralizing GMTs of RSVpreF sera against RSV B clinical isolates.

Clinical isolate ID	Sera samples (n)	GMT (95% CI)	GMR relative to reference strain (95% CI)
B18537 (Ref)	30	37,616 (24,380, 58,038)	1.00
PFERSV012	30	28,793 (19,264, 43,036)	0.77 (0.64, 0.91)
PFERSV014	30	21,451 (14,226, 32,345)	0.57 (0.34, 0.96)
PFERSV018	30	29,662 (19,530, 45,051)	0.79 (0.61, 1.02)
PFERSV020	30	16,500 (11,341, 24,004)	0.44 (0.35, 0.56)
PFERSV022	30	17,136 (11,193, 26,236)	0.46 (0.36, 0.58)
PFERSV277	30	79,613 (53,815, 117,777)	2.10 (1.70, 2.60)
PFERSV292	30	45,056 (31,893, 63,652)	1.20 (0.93, 1.55)
PFERSV293	30	42,745 (30,829, 59,266)	1.14 (0.90, 1.43)
PFERSV294	30	18,630 (13,186, 26,321)	0.50 (0.38, 0.64)
PFERSV297	30	46,658 (33,233, 65,507)	1.24 (0.99, 1.55)
PFERSV301	30	51,113 (35,141, 74,346)	1.36 (1.11, 1.67)
PFERSV387	30	25,576 (17,614, 37,139)	0.68 (0.55, 0.84)
PFERSV389	30	45,150 (31,258, 65,216)	1.20 (0.97, 1.486)
B18537 (Ref)	30	26,762 (17,687, 40,492)	1.00
PFERSV380*	30	20,899 (14,180, 30,801)	0.78 (0.64, 0.96)
PFERSV381*	30	14,695 (9,652, 22,373)	0.55 (0.44, 0.68)
PFERSV386*	30	24,690 (17,078, 35,696)	0.92 (0.75, 1.14)
PFERSV390*	30	28,314 (19,633, 40,833)	1.06 (0.83, 1.34)
PFERSV391*	30	14,694 (10,077, 21,426)	0.55 (0.45, 0.67)
PFERSV393*	30	19,489 (14,123, 26,894)	0.73 (0.59, 0.90)
PFERSV395*	30	25,435 (17,823, 36,298)	0.95 (0.75, 1.20)
PFERSV396*	30	14,723 (10,591, 20,468)	0.55 (0.43, 0.70)
PFERSV397*	30	12,901 (8,519, 19,536)	0.48 (0.40, 0.59)
PFERSV399*	30	21,179 (14,301, 31,367)	0.79 (0.65, 0.97)
All Isolates		26,090 (24,252, 28,067)	0.79 (0.75, 0.83)

GMT, geometric mean titer; GMR, geometric mean ratio; Ref, reference strain
*Post -2020 RSV isolates

Supplementary Table 4. IC_{50} of nirsevimab against select RSV A and B clinical isolates.

RSV Subgroup	Clinical isolate ID	F antigenic site affected	Nirsevimab IC_{50} ng/mL	GMC ng/mL	IC_{50} fold difference vs M37 reference strain
A	M37 (Ref)		5.7		1.0
	PFERSV335	Ø	3.9	5.1	0.7
	PFERSV400	Ø	7.5		1.3
	PFERSV408	V	2.3		0.4
	PFERSV421	II	7.0		1.2
	PFERSV425	II	7.2		1.3
B	B18537 (Ref)		45.8		8.0
	PFERSV012	III/V	16.0	19.4	2.8
	PFERSV014	III/V	24.8		4.4
	PFERSV018	III/V	17.8		3.1
	PFERSV020	III/V	20.1		3.5
	PFERSV277	Ø/III/V	2.5	2.8	0.4
	PFERSV297	Ø/II/III/V	3.1		0.5
	PFERSV301	Ø/III/V	2.4		0.4
	PFERSV381	Ø/III/V	4.0		0.7
	PFERSV391	Ø/I/II/III/V	2.5		0.4

GMC, geometric mean concentration; IC_{50}, 50% maximal inhibitory concentration; Ref, reference strain

Supplementary Table 5. Global prevalence of RSV B genome sequences containing triple I206M/Q209R/S211N, double I206M/Q209R, and single S211N F protein amino acid substitutions in GISAID, 2018-2024.

	Number of RSV B entries[a] (Percentage of total RSV B entries/year)		
**Year**	**I206M/Q209R/S211N**	**I206M/Q209R**	**S211N**
2018	2 (<1%)	1381 (82%)	2 (<1%)
2019	8 (1%)	1396 (94%)	8 (1%)
2020	4 (1%)	454 (96%)	4 (1%)
2021	543 (37%)	1372 (94%)	543 (37%)
2022	1189 (68%)	1695 (97%)	1205 (69%)
2023	1871 (92%)	1975 (97%)	1897 (93%)
2024[a]	977 (94%)	1004 (97%)	1003 (96%)

[a] GISAID query results used July 31, 2024 as the cut-off date

References

1 Graham, B. S. Immunological goals for respiratory syncytial virus vaccine development. *Current opinion in immunology* **59**, 57-64 (2019). https://doi.org:10.1016/j.coi.2019.03.005
